# Sexually dimorphic gene expression responses of bovine embryos to the maternal microenvironment on day 13 of gestation

**DOI:** 10.1186/s12864-025-11570-5

**Published:** 2025-04-14

**Authors:** Dessie Salilew-Wondim, Ernst Tholen, Christine Große-Brinkhaus, Eva Held-Hoelker, Dennis Miskel, Franca Rings, Karl Schellander, Urban Besenfelder, Vitezslav Havlicek, Dawit Tesfaye, Michael Hoelker

**Affiliations:** 1 Animal Breeding, Institute of Animal Sciences, University of Bonn, Endenicher Allee 15, Bonn, 53115 Germany; 2 Department of Animal Sciences, Biotechnology and Reproduction of Farm Animals, University of Göttingen, Burckhardtweg 2, Göttingen, 37077 Germany; 3 Institute of Animal Breeding and Genetics, University of Veterinary Medicine Vienna, Vienna, 1210 Austria; 4 Department of Biomedical Sciences, Animal Reproduction and Biotechnology Laboratory, Colorado State University, 3105 Rampart Rd, Fort Collins, CO 80521 USA

**Keywords:** Sexual-dimorphism, Transcriptome, Elongation, Embryo, Maternal-microenvironment

## Abstract

**Background:**

Various studies have highlighted significant differences in developmental kinetics and sensitivity to developmental conditions between male and female bovine embryos. These differences are thought to be caused in part by the sexually dimorphic expression of genes located on the sex or autosomal chromosomes. However, little is known about the dimorphic gene expression patterns of bovine embryos at the initiation of elongation, which is one of the critical stages of development. Furthermore, to the best of our knowledge, there is little or no data available on the sexually dimorphic gene expression patterns in bovine embryos in relation to maternal environmental conditions during the initiation of elongation. Therefore, the main objective of this study was to investigate the sexually dimorphic gene expression responses of embryos to the maternal environment at the initiation of elongation in embryos developed in lactating dairy cows and nonlactating nulliparous heifers.

**Results:**

Gene expression analysis showed that 159 genes including those involved in steroid biosynthesis and gastrulation were differentially expressed exclusively between male and female embryos developed in cows. Among these, 61 genes including *CYP39 A1, CYP2R1* and *CYP27B1* were upregulated and 98 genes including *HSD17B1*, *HSD17B10* and aromatase *(CYP19 A1)* were downregulated in male embryos. Chromosomal analysis showed that 31.2% of the differentially expressed genes (DEGs) including glucose-6-phosphate dehydrogenase (*G6PD)* were located on the X chromosome, and 96% of those were upregulated in female embryos. Similarly, 254 genes including those involved in female sex differentiation, placenta development, transmembrane transport, and cell adhesion were differentially expressed exclusively between the male and female embryos developed in heifers. Of these, 108 genes including *HSD17B11*, *HSD17B12,* and *HSD3B1* were upregulated, and 146 genes including *SLC16 A9, SLC10 A1, SLC10 A3, SLC16 A5, SLC22 A23, SLC25 A43, SLC35 A2, SLC35 C1,* and *SLC4* were downregulated in male compared to female embryos. In addition, 17.3% of the DEGs were located on the X chromosome and 75% of the DEGs located on the X chromosome were upregulated in female embryos. On the other hand, 38 genes including *SLC30 A10, SLC10 A4, ATP6 AP1,* and *KDM5 C* showed sexually dimorphic expression patterns in day 13 bovine embryos irrespective of the maternal environment. These genes accounted for only 19% and 13% of the genes that showed sexually dimorphic expression in embryos developed in cows and heifers, respectively and the expression difference of these genes in male and female embryos was then likely influenced by the sex of the embryo.

**Conclusion:**

This study revealed that embryos developed in lactating cows showed sexually dimorphic expression of genes involved in various functions including steroid biosynthesis and gastrulation. In contrast, embryos developed in heifers displayed sexually dimorphic expression of genes related to placental development, female sex differentiation, and transmembrane transport. This suggests that the reproductive tract environments of cows and heifers differently affect the sex specific expression of genes in bovine embryos. A higher proportion of genes that showed sexually dimorphic expression in cow embryos were located on the X chromosome, and the majority of these genes were upregulated in female embryos. Overall, this study provides insight into genes that exhibit sexually dimorphic expression patterns in day 13 bovine embryos due to the maternal reproductive tract microenvironment or solely due to the sex of the embryo.

**Supplementary Information:**

The online version contains supplementary material available at 10.1186/s12864-025-11570-5.

## Introduction

Although most sexual dimorphisms in embryos are expected to appear after gonadal differentiation, differences between male and female bovine embryos in their developmental kinetics and responses to environmental conditions [1–4] occur at the early stage of development prior to gonadal differentiation. For instance, sex specific difference in glucose metabolism was observed in day 7 embryos [5], and the supplementation of glucose in culture media promoted male embryos to cleave faster than female ones [6] and more male morula embryos to progress to advanced stages at a higher rate than the female ones [7, 8]. On the other hand, oxidative culture conditions reduced cell numbers and increased apoptotic cells in female blastocysts [9, 10]

Although tracing and identifying the relevant factors that contribute to the differences in the developmental kinetics and sensitivity to culture conditions of the male and female embryos is a focus of reproduction research, the sexually dimorphic expression of genes located on sex chromosomes or autosomes is believed to be one of the factors that contribute to these phenomena [4, 11–15]. For instance, the upregulation of glucose- 6-phosphate dehydrogenase (*G6PDH*) [8, 16, 17] and X inactive specific transcript (*Xist*) [17] has been shown in in vitro produced female blastocysts. Moreover, Forde et al. [18] have also indicated sexually dimorphic expression of several genes including those involved in cell cycle progression, DNA methylation and transcriptional repression signaling, and the mTOR signaling pathway in day 19 bovine embryos. Findings from that study were generated after analyzing fully in vivo derived bovine embryos at the initiation of implantation in one developmental environment (heifers). However, a comparison of in vitro produced day 2 embryos transferred into animals in different physiological conditions, in terms of their sexually dimorphic gene expression at the early elongation has not been performed.

In bovine embryos, the transformation of blastocysts from a spherical to a filamentous structure occurs around day 13 of gestation [19–21] and this period is crucial for in bovine embryonic developmental process [22] as it marks the initiation of embryo elongation [23]. Several genes associated with the transition from a spherical blastocyst to an ovoid conceptus or genes associated with the initiation of embryo elongation were expressed in day 13 embryos [23]. Around this period, the elongating embryo modulates the endometrium transcriptional activities in an embryo sex-specific manner [21]. However, information on sexually dimorphic gene expression patterns in day 13 embryos, which coincides with the initiation of elongation, is lacking. Thus, further study is needed to gain a better understanding of the sexually dimorphic gene expression patterns during this critical period. To the best of our knowledge, currently, there is little or no data available on the gene expression profiles of male and female embryos in relation to developmental conditions during the initiation of embryo elongation, particularly on day 13 of gestation. It is also unclear whether male and female embryos share common gene expression patterns during the initiation of elongation when they develop under different conditions. Moreover, embryo losses are more frequent in lactating postpartum cows than in nonlactating nulliparous heifers [24, 25]. Since the maternal reproductive tract environments are major players in determining developmental kinetics and embryo survival [26], the question arises whether the reproductive tract environment of a cow and heifer could differentially modulate the expression of genes in male and female embryos. Indeed, during development, the embryos are believed to respond to environmental stresses by modulating critical events including X chromosome inactivation [27] and switching off/on sex specific expression of transcriptional factors [10]. Therefore, these and other findings highlighted the need for further studies to understand whether the contrasting maternal environmental conditions can cause the male and female embryos to respond differently. This could be explained by analyzing the sexually dimorphic gene expression during the early elongation of embryos. Therefore, taking all these factors into account, we hypothesized that during the critical period of development, male and female embryos respond differently to the maternal environment through sex-specific modulation of their transcriptional activity. Therefore, the main objective of this study was to investigate the sexually dimorphic gene expression responses of bovine embryos to the maternal environment during the initiation of elongation. To achieve the objective of this study, we used two groups of embryos (male and female), and two groups of recipients: lactating Holstein Friesian dairy cows which are assumed to provide a less ideal or metabolically demanding maternal environment, and heifers which are thought to provide a nearly ideal developmental environment. Accordingly, we first identified the genes expressed in day 13 male and female embryos developed in lactating postpartum cows and nulliparous heifers. Secondly, we investigated the sexually dimorphic gene expression patterns in day 13 embryos developed in cows and heifers. Finally, we identified genes that showed sexually dimorphic expression patterns in day 13 embryos irrespective of the maternal reproductive tract microenvironment.

## Materials and methods

### Animal handling and management

Experimental animals (Holstein Frisian lactating cows and Holstein Frisian nulliparous heifers) were kept at the Frankenforst research station of the University of Bonn. Handling and management of experimental animals adhered to the rules and regulations of the German law of animal protection. The experiment involving animals was approved by the Animal Welfare (ethics) committee of the University of Bonn and the Federal Ministry of Food and Agriculture of Germany, with preposition number 84–02.04.20.2015.A083. For embryo sample collection, animals were slaughtered at a local slaughterhouse under the supervision of the responsible local veterinary office. Animals were first stunned with a bolt stunner prior to final slaughter.

### In vitro production of male and female embryos, endoscopic tubal transfer of embryos, and recovery of day 13 embryos

In vitro maturation, in vitro fertilization, in vitro culture, tubal embryo transfer of day 2 embryos, recovery and classification of day 13 embryos have been described in detail in our previous publication [28]. Briefly, cumulus-oocyte complexes (COCs) were collected from slaughterhouse ovaries. After in vitro maturation, oocytes were divided into two groups and in vitro fertilized with X—or Y-chromosome-bearing sperm from the Holstein Frisian bull. In vitro fertilization was performed in Fert-TALP medium supplemented with 20 μM penicillamine, 10 μl PHE (Hypotaurine-Epinephrin-solution), 6 mg/ml BSA-FFA, 50 μg/ml gentamycin, and 10 μg/ml heparin. The final sperm concentration in fertilization droplets was adjusted to 2 × 10^6^ sperm/ml. After co-incubation of COCs with sperm cells for 18 h, the cumulus and sperm cells were removed. The two zygote groups (male and female) were then in vitro cultured in groups of 50 in 400 μl of synthetic oviductal fluid (SOF) supplemented with 0.6% fatty acid-free bovine serum albumin (BSA) under 5% O_2_, 5% CO_2_ and 39 °C until day 2. Afterwards, day 2 embryos were transferred to oestrus synchronized cows and heifers. For this purpose, Holstein Friesian heifers (*n* = 11) with no history of calving and lactating Holstein Friesian cows at 50–120 days postpartum (*n* = 10) in parity 1–2 were oestrus synchronized by two intramuscular administration of 500 mg of cloprostenol (Estrumate; Munich, Germany) at 11 days interval. Gonadotropin releasing hormone (GnRH) (Receptal; Intervet, Boxmeer, The Netherlands) was administered after each cloprostenol injection. Following this, lactating cows (*n* = 6) and heifers (*n* = 6) each received 20 female embryos, while other cows (*n* = 4) and heifers (*n* = 5) each received 20 male embryos using endoscopic embryo tubal transfer on day 2 of the oestrous cycle. Embryos were transferred unilaterally ipsilateral to the corpus luteum after careful observation using ultrasonography. Embryos were then recovered on day 13 of the gestation after slaughtering. The recovered embryos were then classified into four groups. Male and female embryos developed in lactating cows were classified as CM and CF groups, respectively, and male and female embryos developed in heifers were classified as HM and HF groups, respectively. All samples (CM, CF, HM, and HF) were initially snap frozen in liquid nitrogen and stored at − 80 ^O^C until further analysis.

### RNA isolation from male and female embryos

Since this study is a continuation of our previous work, the RNA extraction method used for CM, CF, HM and HF samples has been described in our previous publication [28]. Briefly, the AllPrep DNA/RNA/miRNA universal kit was used to isolate total RNA from individual embryos. Each embryo was lysed in lysis buffer containing β-mercaptoethanol (1%) and passed through the QIAshredder (Qiagen) by centrifugation at maximum speed for 2 min. DNA was removed using the DNA spin column and protein was removed by incubating the flowthrough containing RNA with 50 μl proteinase K. The sample was then transferred to an RNeasy® Spin Column fitted with a 2 ml collection tube and centrifuged for 15 s. After sequential washes in 500 μl Buffer RPE and 500 μl of 96–100% ethanol, any remaining DNA was removed by performing on-column DNA digestion. After subsequent steps, total RNA was eluted in 35 μl RNase-free water. RNA quality was evaluated using Agilent 2100 bioanalyzer integrated with the RNA 6000 Nano LabChip® Kit (Agilent Technologies Inc, CA, USA). The amount of RNA was determined using a Nanodrop 8000 spectrophotometer (Thermo Fisher Scientific Inc, DE, USA). A total of 20 RNA samples (5 RNA samples per experimental group) with RNA integrity number (RIN) ≥ 6, A260/A280 = 1.8—2.2 and a total concentration of ≥ 500 ng were used for RNA sequencing.

### Library preparation with polyA selection and Sequencing

RNA library preparation and sequencing were performed by Azenta Life Sciences (South Plainfield, NJ, USA). Briefly, prior to library preparation, sample RNA concentrations were quantified using the Qubit 2.0 Fluorometer (Life Technologies, Carlsbad, CA, USA), and RNA integrity was evaluated using the Agilent TapeStation 4200 (Agilent Technologies, Palo Alto, CA, USA). NEBNext Ultra II RNA Library Prep (NEB, Ipswich, MA, USA) was used to prepare the sequencing libraries. Briefly, the mRNAs were enriched with Oligod(T) beads, fragmented at 94 °C for 15 min, and then first- and second-strand cDNAs were synthesized. After end repair, the cDNA fragments were adenylated at the 3’ end, followed by universal adapter ligation, index addition and library enrichment by PCR. The library from each sample was then validated using the Agilent TapeStation (Agilent Technologies, Palo Alto, CA, USA) and quantified using the Qubit 2.0 Fluorometer (Invitrogen, Carlsbad, CA) and quantitative PCR (KAPA Biosystems, Wilmington, MA, USA). Libraries were clustered on the flowCell and loaded on the Illumina instrument (4000 or equivalent) following the manufacturer’s instructions. Sequencing was done in a paired-end (PE) configuration. Raw sequence data (.bcl files) were converted to fastq files and de-multiplexed using Illumina's bcl2fastq software (version 2.17). Raw and processed data RNA seq data are available in the NCBI repository: https://www.ncbi.nlm.nih.gov/geo/query/acc.cgi?acc=GSE276275.

### Adapter trimming and sequence mapping to the bovine reference genome

The quality of the raw data was evaluated using FastQC, a freely available sequence analysis tool, (http://www.bioinformatics.babraham.ac.uk/publications.html). Adapters, PCR primers and non-informative sequences were removed from downstream analysis using trim galore software (Babraham Bioinformatics). Prior to mapping the reads, the bovine reference genome (Bos_taurus.ARS-UCD1.2) was indexed using bowtie2-build. Sequences were then mapped to the reference genome using the bowtie2 tool by setting paired-end and end-to-end read alignment parameters. The sequence alignment map (SAM) files from the read alignments were then converted to the binary alignment map (BAM) files and sorted using the Samtools command [29]. Sorted BAM files were used for downstream analysis.

### Identification of expressed and differentially expressed genes

Sorted BAM files were imported into the Seqmonk tool (version 1.48.0, Babraham Bioinformatics). Reads with primary alignments and mapping quality ≥ 20 were imported. Quantitation was performed using the RNA-Seq pipeline option of Seqmonk. Following quantification, expressed genes in each sample were identified and annotated. Genes with read counts of 9500 or more were considered to be highly expressed genes.

Differential gene expression analysis was done using the edgeR Bioconductor package [30]. Briefly, library sizes were normalized between samples by applying a set of scaling factors using a trimmed mean of M values (TMM). Quantile-adjusted conditional maximum likelihood common dispersion and tagwise dispersion were estimated using the estimateDisp function. Finally, the mean expression differences between samples were tested using the exatTest function. Differentially expressed genes were screened based on absolute fold change ≥ 1.5, p-value < 0.05 and the false discovery rate (FDR) < 0.1.

### Gene ontology enrichment analysis

Gene ontology enrichment analysis was performed using g:profiler tool [31], a web-based tool for functional enrichment analysis. Gene set enrichment analysis was performed by considering all known bovine genes. Significant gene set enrichments were filtered based on the Benjamini–Hochberg false discovery rate (FDR).

Cytoscape (version 3.10.0), a general-purpose modelling environment for integrating biomolecular interaction networks and states [32] was used to build a network between differentially expressed genes and biological processes. For this, a file containing biological processes and gene lists with their respective fold changes was prepared and imported into Cytoscape (version v3.10.0). Biological processes were designated as the sources. The height, width and transparency of the nodes were set to 100, 200, and 250, respectively. The fill colour of the upregulated and downregulated genes was set to red and sky, respectively. The networks were exported as images.

## Results

### Genes expressed in day 13 embryos

Embryo recovery rates including degenerated embryos on day 13 of gestation were 58.8%, 46.7%, 59.0%, and 59.2%, in the CM, CF, HM, and HF groups, respectively. Excluding degenerated embryos, the recovery rates were 42.5%, 15.0%, 28.0% and 26.7%, in CM, CF, HM and HF groups, respectively (Table 1).

**Table 1:** The number of day 2 embryos transferred and day 13 embryo recovery rates

Embryo group	Recipient	Number of embryos transferred	Total embryo recovery rate (%)	Viable embryo recovery rate (%)
Male	Cow	80	58.8	42.5
Female	Cow	120	46.7	15.0
		**200**	**51.5**	**26.0**
Male	Heifer	100	59.0	28.0
Female	Heifer	120	59.2	26.7
		**220**	**59.1**	**27.2**

Modified from Salilew-Wondim et al. [28]

A gene expression study was then performed in CM, CF, HM and HF groups disregarding the degenerated embryos. Accordingly, 40 RNA sequence data (20 reverse and 20 forward sequence data) with 150 bp paired-end sequences were generated from 20 libraries. Regarding read counts, on average, 22.3, 21.5, 22.5, and 22.1 million paired-end reads with 6.7, 6.5, 6.7, and 6.6 Giga bases of sequences with 51% GC content were generated in each biological replicate sample of CM, CF, HM, and HF, respectively. After adapter and non-informative sequence trimming, on average 6.2, 5.9, 6.2, and 6.2 Giga base sequences in CM, CF, HM and HF samples, respectively remained for downstream analysis. The average mapping rates in the CM, CF, HM, and HF groups were 72.1, 72.8, 73.2 and 73.2%, respectively.

**Fig. 1:**
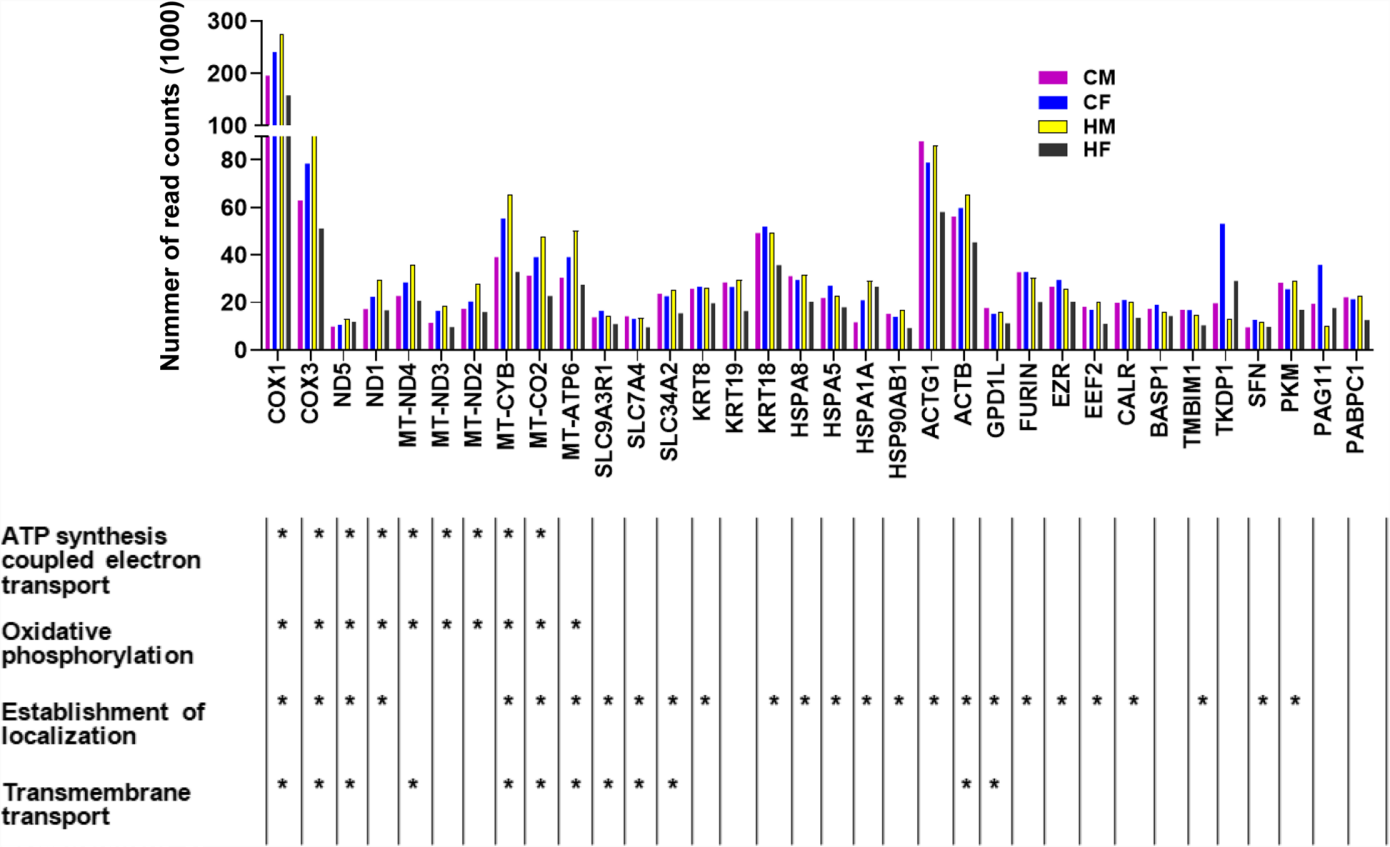
Expression patterns of the highly expressed genes and their functional characterization in day 13 male and female embryos developed in cows or heifers

Gene detection analysis indicated that 12,167, 12,021, 12,180, and 11,465 genes showed ≥ 5 read counts in the CM, CF, HM and HF groups, respectively. Including the mitochondrial genes (*MT-ND4, MT-ND3, MT-ND2, MT-CYB, MT-CO2, MT-ATP6, COX3, COX1*), keratins (*KRT8, KRT18, KRT19*), heat shock proteins (*HSPA8, HSPA5, HSPA1 A, HSP90 AB1*), 34 annotated genes showed relatively higher read counts (9500—274,090) in all embryo groups. Based on their read counts, these genes were considered to be the most highly expressed genes in the day 13 bovine embryos (Fig. 1). The highly expressed genes were involved in ATP synthesis coupled electron transport, oxidative phosphorylation, establishment of localization and transmembrane transport biological process (Fig. 1).

### Sexually dimorphic gene expression patterns in embryos developed in lactating postpartum cows

**Fig. 2:**
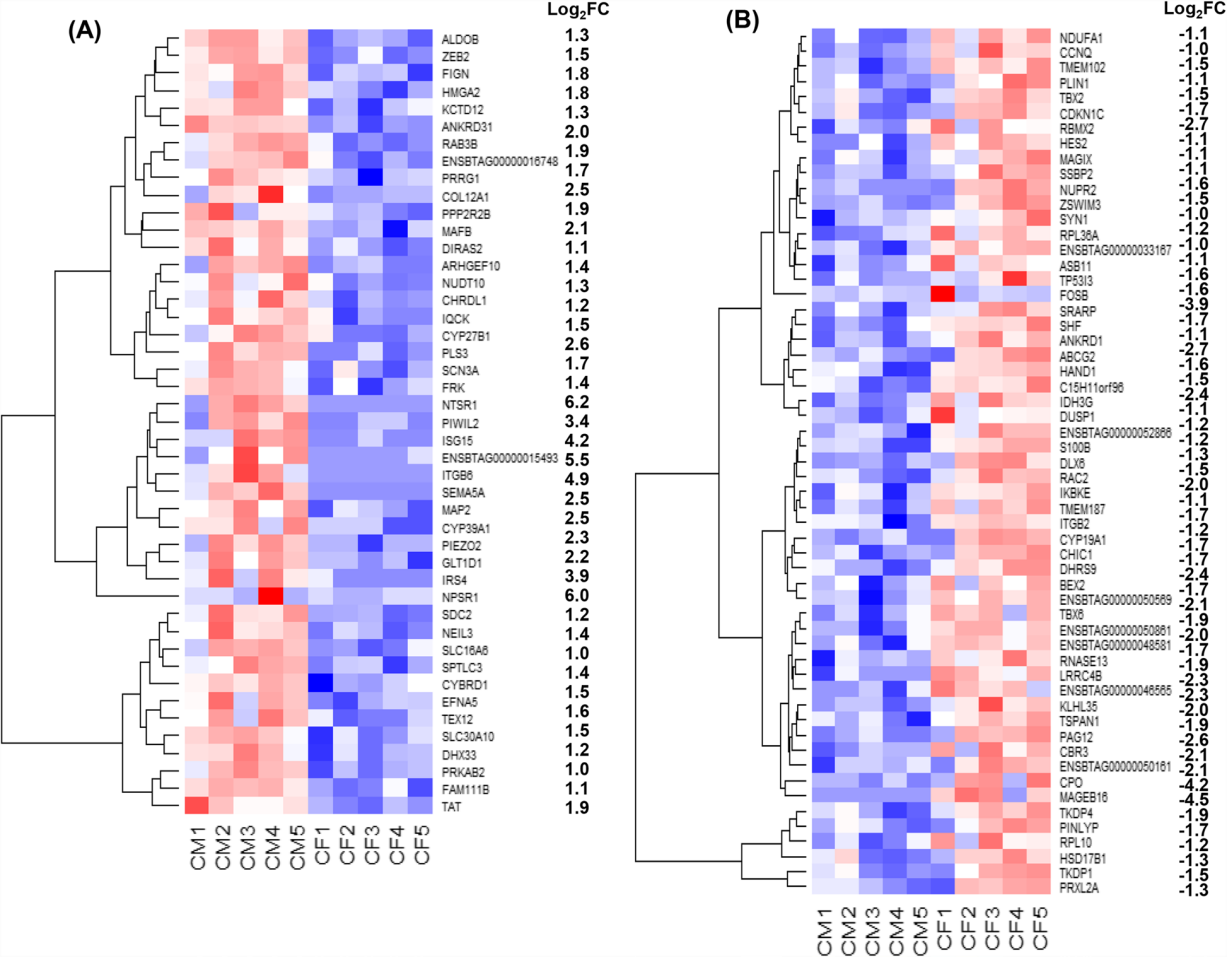
Heatmap indicating the expression patterns of genes upregulated with a log_2_FC of ≥ 1 (A) or downregulated with a log_2_FC of ≤ − 1 (B) in the CM compared to the CF embryo group. The Red and blue colours indicate increased and decreased gene expression, respectively, in the CM compared to the CF group. CM1, CM2, CM3, CM4, and CM5 represent biological replicates in the CM embryo group and CF1, CF2, CF3, CF4, and CF5 represent biological replicates in the CF embryo group. Log_2_FC: log_2_ fold change

**Fig. 3:**
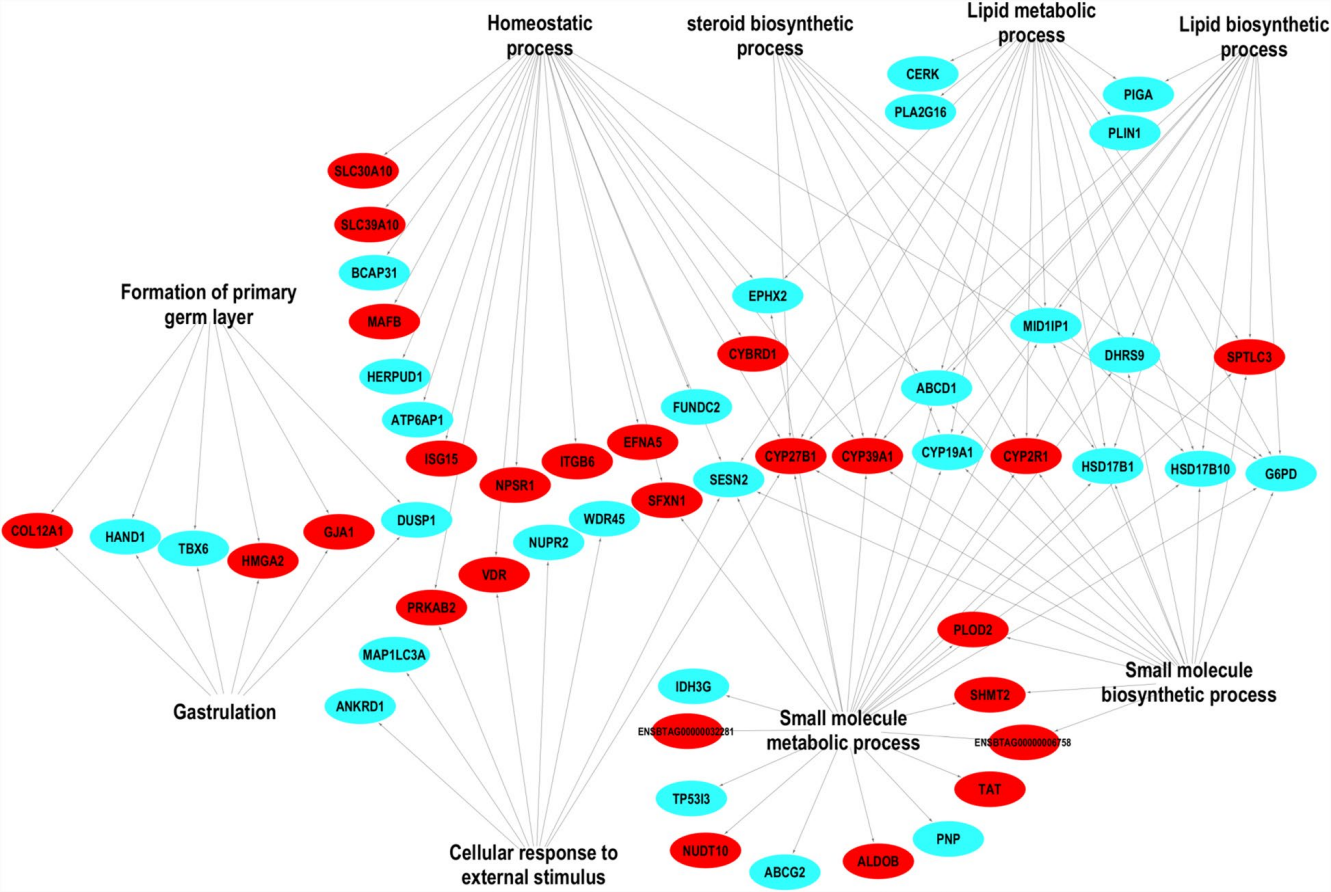
Biological processes enriched by differentially expressed genes between the CM and CF groups. Red and sky colors indicate increased and decreased gene expression, respectively

To explore the sexually dimorphic gene expression patterns in embryos developed in lactating cows, we compared the transcriptome profiles of the male (CM) and female (CF) embryos. The results showed that 197 genes were differentially expressed between these embryo groups, of which 68 DEGs showed increased expression, while 129 DEGs showed decreased expression in CM compared to CF (Figure S1). Among the DEGs, the expression levels of *NTSR1, NPSR1, ITGB6, SEMA5 A, IRS4, ISG15, DIRAS2* and *PIWIL2* were increased, whereas the expression levels of *MAGEB16, CPO* and *FOSB* were decreased by at least by 4 folds in CM compared to CF (Fig. 2). Moreover, the family of genes including cytochrome P450 (*CYP2R1, CYP27B1, CYP39 A1*) and solute carriers (*SLC10 A4, SLC16 A1, SLC16 A6, SLC30 A10, SLC39 A10*) were upregulated, while ribosomal proteins (*RPL10, RPL36 A, RPL39*) were downregulated in the CM compared to the CF embryo groups (Table 2). Gene enrichment analysis showed that genes differentially expressed between CM and CF were involved in gastrulation, steroid biosynthesis and metabolism, lipid metabolism, and homeostasis (Fig. 3).

**Table 2:** Differentially expressed gene families between CM and CF embryos

Gene symbol	Ensembl gene id	Log_2_ FC	P value	FDR
SLC10 A4	ENSBTAG00000004888	0.7	4.98E- 05	0.015
SLC16 A1	ENSBTAG00000015107	0.6	5.12E- 04	0.072
SLC16 A6	ENSBTAG00000003074	1.0	7.69E- 05	0.019
SLC30 A10	ENSBTAG00000004414	1.5	4.41E- 05	0.014
SLC39 A10	ENSBTAG00000016782	0.7	7.73E- 04	0.090
CYP19 A1	ENSBTAG00000014890	− 1.7	1.45E- 04	0.029
CYP27B1	ENSBTAG00000016906	1.5	4.15E- 04	0.064
CYP2R1	ENSBTAG00000010419	0.9	8.60E- 04	0.097
CYP39 A1	ENSBTAG00000003632	2.4	7.01E- 04	0.085
RPL10	ENSBTAG00000007454	− 1.2	8.39E- 06	0.005
RPL36 A	ENSBTAG00000019253	− 1.0	2.67E- 04	0.049
RPL39	ENSBTAG00000049833	− 0.9	5.45E- 05	0.016

*Log*_*2*_* FC, log*_*2*_* fold changes*

### Sexually dimorphic gene expression patterns in day 13 embryos developed in heifers

**Fig. 4:**
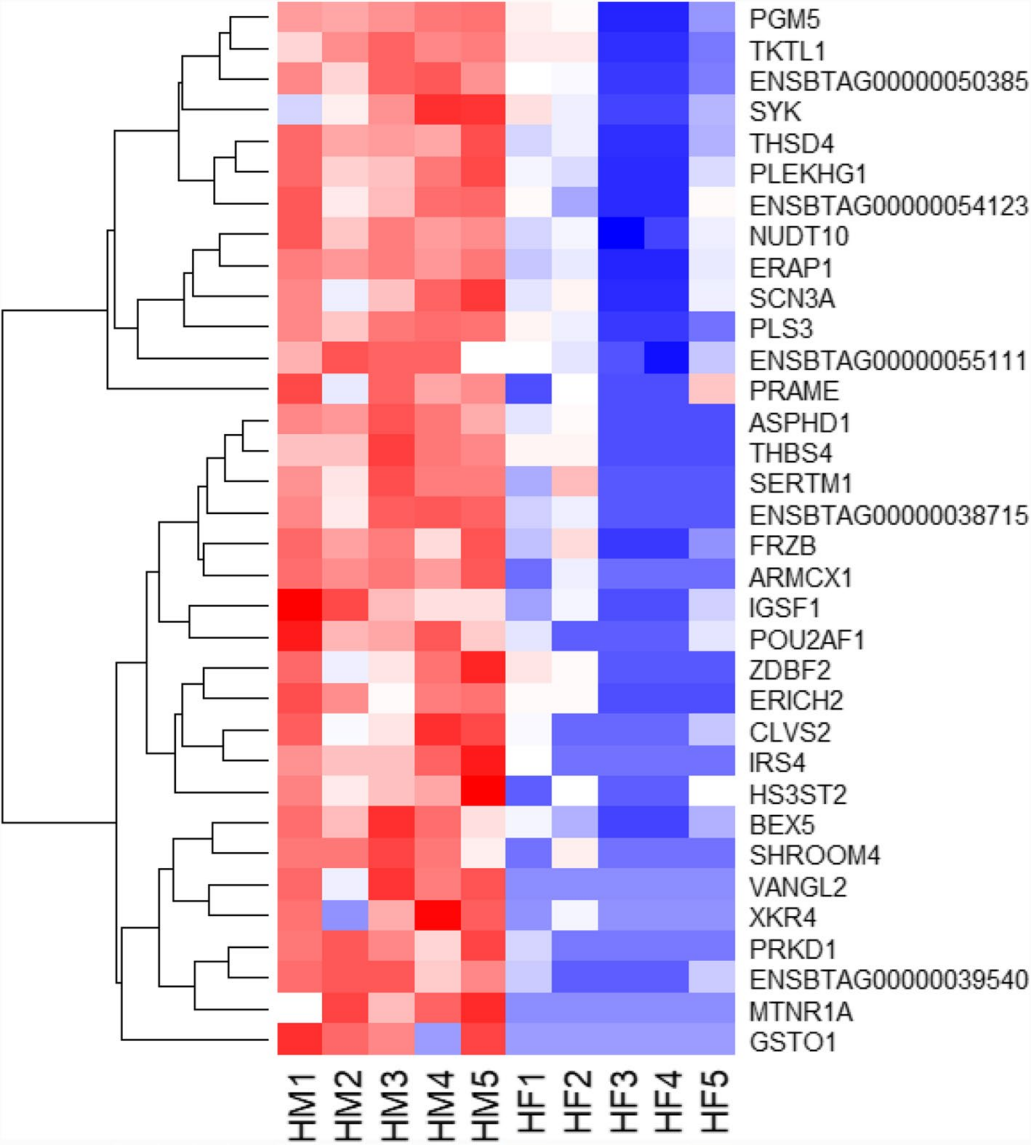
The expression patterns of genes significantly upregulated with a log_2_FC of ≥ 2 in HM compared to the HF embryo group. HM1, HM2, HM3, HM4, and HM5 represent biological replicates in the HM embryo groups and HF1, HF2, HF3, HF4, and HF5 represent biological replicates in the HF embryo group. Red and blue colours indicate increased and decreased gene expression, respectively

**Fig. 5:**
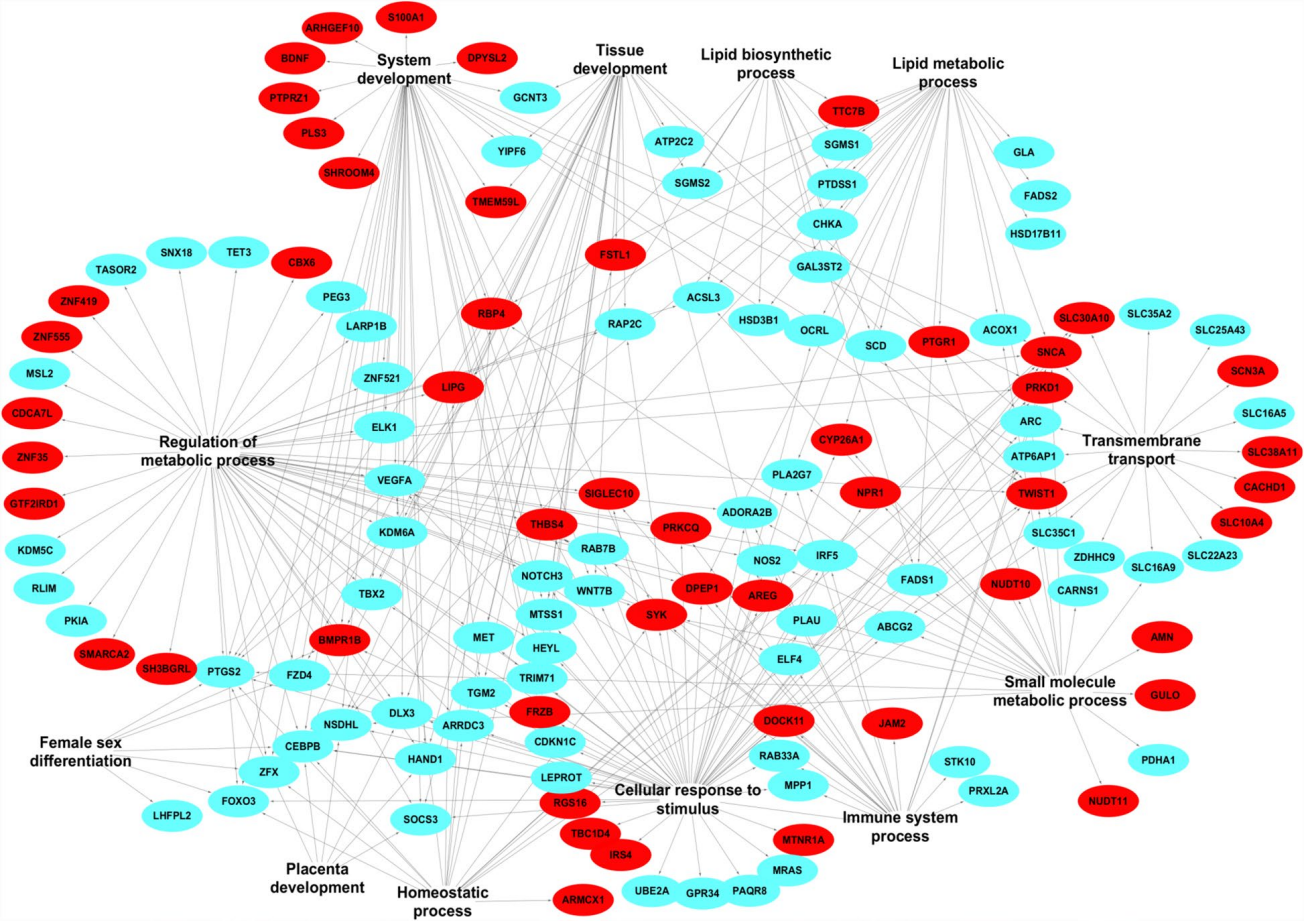
Biological processes enriched by differentially expressed genes between HM and HF. Red and sky colors indicate the upregulated and downregulated genes, respectively, in HM compared to HF

To investigate the sexually dimorphic expression of genes in male and female embryos at the initiation of elongation in relation to the reproductive tract microenvironment of heifers, we analyzed the gene expression profiles of male (HM) and female (HF) embryos developed in heifers. The results showed that 115 and 177 genes were upregulated and downregulated, respectively in HM compared to the HF embryos (Figure S2). Among these, 34 DEGs including *GSTO1, VANGL2, MTNR1 A, PRKD1,* and *ARMCX1* showed at least fourfold higher expression (Fig. 4), and 26 DEGs including *NOS2, ARC, DHRS9*, and *PTGS2* showed at least fourfold lower expression in the HM compared to the HF group (Table 3). Moreover, gene families including glucosaminyl (N-acetyl) transferases, hydroxysteroids*,* solute carriers, ATPases, and zinc finger proteins were differentially expressed between the two embryo groups (Table 4). Gene enrichment analysis revealed that genes differentially expressed between these two groups of embryos were involved in tissue development, system development, female sex differentiation, placenta development, regulation of metabolic processes, and cellular response to stimulus (Fig. 5).

**Table 3:** List of genes downregulated by ≥ fourfold changes in HM compared to HF

Gene symbol	Ensembl gene id	Log_2_ FC	P value	FDR
HSD3B1	ENSBTAG00000006769	− 2.2	2.32E- 12	0.0000
GPR34	ENSBTAG00000020144	− 2.0	2.25E- 12	0.0000
SERPINE1	ENSBTAG00000014465	− 2.6	1.67E- 09	0.0000
TKDP4	ENSBTAG00000013950	− 2.5	1.60E- 08	0.0000
TBX2	ENSBTAG00000014278	− 2.7	8.58E- 08	0.0002
PKD2L1	ENSBTAG00000010742	− 3.0	1.01E- 07	0.0002
PTGS2	ENSBTAG00000014127	− 3.4	1.19E- 06	0.0011
LARP1B	ENSBTAG00000012135	− 2.0	2.03E- 06	0.0018
ENSBTAG00000049445	ENSBTAG00000049445	− 2.1	2.23E- 06	0.0018
PINLYP	ENSBTAG00000001260	− 3.0	3.79E- 06	0.0025
PAQR8	ENSBTAG00000025494	− 2.6	6.26E- 06	0.0033
ENSBTAG00000039337	ENSBTAG00000039337	− 2.5	6.18E- 06	0.0033
CDKN1 C	ENSBTAG00000031184	− 2.3	6.92E- 06	0.0034
RAB7B	ENSBTAG00000012774	− 2.1	1.36E- 05	0.0058
CAND2	ENSBTAG00000015273	− 2.2	2.85E- 05	0.0105
STK10	ENSBTAG00000017457	− 2.6	3.48E- 05	0.0116
DHRS9	ENSBTAG00000004557	− 3.7	3.92E- 05	0.0126
NOS2	ENSBTAG00000006894	− 5.5	6.95E- 05	0.0183
CNR2	ENSBTAG00000019371	− 2.5	8.44E- 05	0.0210
C15H11orf96	ENSBTAG00000045822	− 2.2	9.72E- 05	0.0225
ARC	ENSBTAG00000021639	− 3.9	1.30E- 04	0.0261
TCHHL1	ENSBTAG00000015854	− 2.6	1.96E- 04	0.0343
ABCG2	ENSBTAG00000017704	− 2.0	2.25E- 04	0.0372
ENSBTAG00000053661	ENSBTAG00000053661	− 6.6	4.79E- 04	0.0599
PLA2G7	ENSBTAG00000019315	− 2.4	8.85E- 04	0.0807
ENSBTAG00000052396	ENSBTAG00000052396	− 3.1	9.38E- 04	0.0837

*Log*_*2*_* FC, log*_*2*_* fold changes*

**Table 4:** Differentially expressed gene families between HM and HF embryos

Gene symbol	Ensembl gene id	Log_2_ FC	P value	FDR
ZNF555	ENSBTAG00000008397	1.6	1.14E- 03	0.095
ZNF521	ENSBTAG00000007383	− 1.6	1.07E- 04	0.023
ZNF419	ENSBTAG00000017613	0.9	6.62E- 04	0.072
ZNF35	ENSBTAG00000034005	1.0	7.22E- 04	0.073
SLC48 A1	ENSBTAG00000032331	− 0.9	2.10E- 04	0.035
SLC38 A11	ENSBTAG00000007650	1.5	3.74E- 06	0.003
SLC35 C1	ENSBTAG00000003199	− 1.3	9.91E- 06	0.005
SLC35 A2	ENSBTAG00000002768	− 0.7	1.05E- 03	0.090
SLC30 A10	ENSBTAG00000004414	1.6	1.22E- 03	0.097
SLC2 A4	ENSBTAG00000009190	1.0	3.22E- 04	0.047
SLC25 A43	ENSBTAG00000001007	− 0.8	2.31E- 04	0.037
SLC22 A23	ENSBTAG00000010943	− 1.0	5.77E- 04	0.066
SLC16 A9	ENSBTAG00000019792	− 1.2	5.62E- 04	0.065
SLC16 A5	ENSBTAG00000001110	− 1.3	3.70E- 04	0.050
SLC10 A4	ENSBTAG00000004888	1.4	1.09E- 04	0.024
SLC10 A3	ENSBTAG00000014333	− 1.0	3.40E- 05	0.012
SLC10 A1	ENSBTAG00000001881	− 1.5	8.27E- 07	0.001
RAB9 A	ENSBTAG00000010923	− 0.7	3.65E- 04	0.049
RAB7B	ENSBTAG00000012774	− 2.1	1.36E- 05	0.006
RAB33 A	ENSBTAG00000034712	− 1.3	3.26E- 06	0.002
NUDT11	ENSBTAG00000005252	1.6	4.96E- 04	0.060
NUDT10	ENSBTAG00000054383	2.2	8.83E- 07	0.001
MYO1B	ENSBTAG00000011256	− 0.7	4.56E- 04	0.058
MYH14	ENSBTAG00000002580	1.0	1.11E- 03	0.093
KDM6 A	ENSBTAG00000003740	− 1.0	1.16E- 06	0.001
KDM5 C	ENSBTAG00000014943	− 0.6	1.21E- 04	0.025
HSD3B1	ENSBTAG00000006769	− 2.2	2.32E- 12	0.000
HSD17B12	ENSBTAG00000000087	− 0.9	7.05E- 04	0.073
HSD17B11	ENSBTAG00000006307	− 0.7	1.51E- 04	0.029
GCNT4	ENSBTAG00000050091	− 1.2	1.12E- 04	0.024
GCNT3	ENSBTAG00000009443	− 1.4	1.72E- 06	0.002
FADS2	ENSBTAG00000015505	− 1.1	3.51E- 06	0.002
FADS1	ENSBTAG00000022294	− 1.0	6.83E- 04	0.073
COL4 A5	ENSBTAG00000014575	1.7	2.76E- 04	0.041
COL18 A1	ENSBTAG00000023907	0.8	7.15E- 04	0.073
ATP6 AP2	ENSBTAG00000017801	− 0.7	6.80E- 04	0.073
ATP6 AP1	ENSBTAG00000012117	− 0.6	2.86E- 04	0.042
ATP2 C2	ENSBTAG00000000945	− 1.1	4.95E- 04	0.060
ATP13 A4	ENSBTAG00000016305	0.9	8.97E- 04	0.081
ARMCX2	ENSBTAG00000019417	1.9	8.53E- 05	0.021
ARMCX1	ENSBTAG00000026139	3.7	8.45E- 07	0.001

*Log*_*2*_* FC, log*_*2*_* fold changes*

### Sexually dimorphic gene expression in day 13 embryos due to the reproductive tract environment of lactating cows

**Fig. 6:**
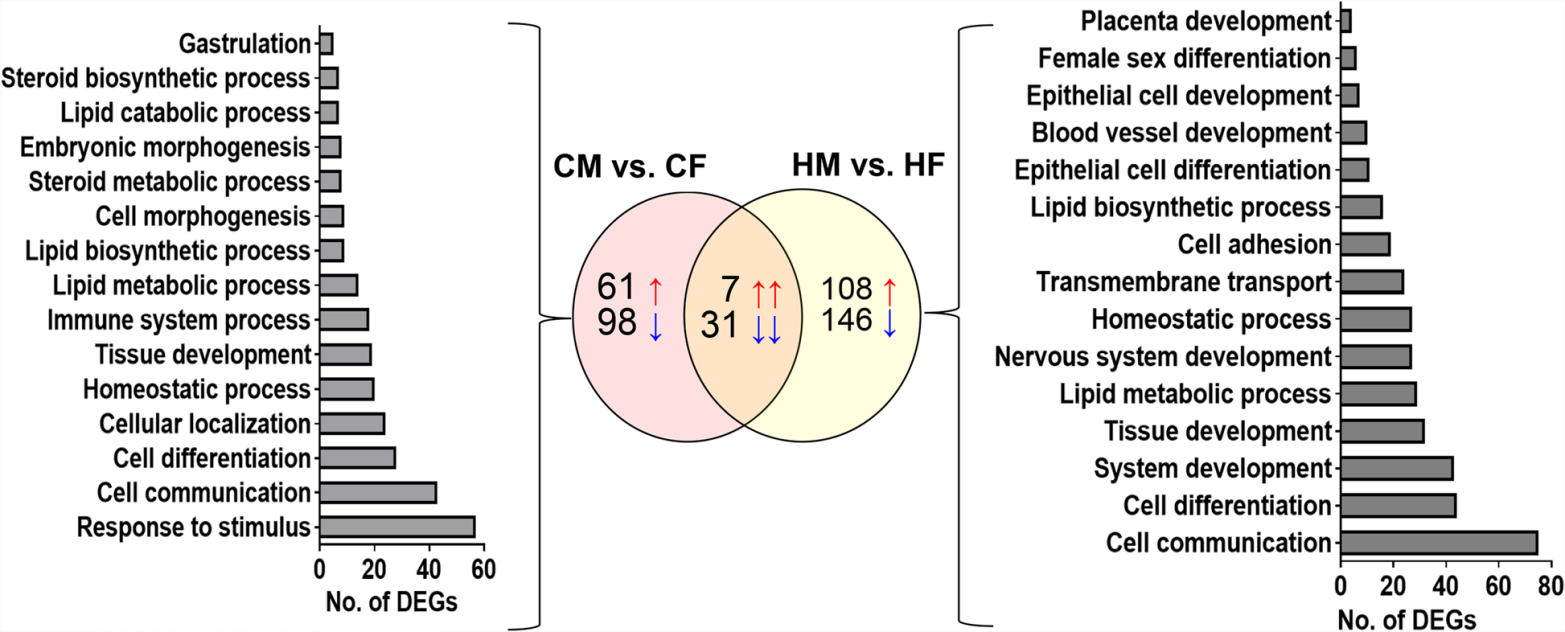
Exclusively differentially expressed genes in CM vs. CF or HM vs. HF comparisons, and genes differentially expressed in both comparisons. The numbers in the circles indicate the number of differentially expressed genes and the upward arrow (↑) and downward arrow (↓) next to each number indicate the upregulation and downregulation of genes, respectively, in CM vs. CF or HM vs. HF. DEGs: Differentially expressed genes

After analyzing sexually dimorphic gene expression in embryos developed in cows or heifers, we identified genes that showed sexually dimorphic expression exclusively in embryos developed in cows using Venny 2.1 software, which compares various lists with Venn diagrams (https://bioinfogp.cnb.csic.es/tools/venny/). The result showed that 159 genes were differentially expressed exclusively between CM and CF, but not between HM and HF (Fig. 6, Table S1). Of these, 61 genes including *CYP39 A1, CYP2R1* and *CYP27B1* were upregulated, whereas 98 genes including *HSD17B1*, *HSD17B10* and aromatase *(CYP19 A1)* were downregulated in CM embryo groups (Table S1). Moreover, 31.2% of those DEGs (Figure S3 A) including glucose- 6-phosphate dehydrogenase (*G6PD*) were located on the X chromosome (Table S1) and, except two genes, all DEGs located on the X chromosome were downregulated in the CM group compared to the CF group, or, upregulated in the CF group compared to the CM group. In silico functional characterization showed that genes that showed sexually dimorphic expression exclusively between male and female embryos developed in cows CM and CF were involved in steroid biosynthesis and metabolism, gastrulation, homeostasis, immune system, and lipid biosynthesis and metabolism (Fig. 6).

### Sexually dimorphic gene expression in day 13 embryos due to the reproductive tract environment of heifers

Using a similar approach to that used in embryos developed in cows, genes that exhibited sexually dimorphic expression solely due to the reproductive tract environment of heifers were identified. The result showed that 254 genes were differentially expressed exclusively between HM and HF (Fig. 6). Among these, 108 genes (57.8% of DEGs) including *SLC2 A4, SLC38 A11, HSD17B11*, *HSD17B12,* and *HSD3B1* were upregulated and 146 genes (42.2% of DEGs) including *SLC10 A1, SLC10 A3, SLC16 A5, SLC16 A9, SLC22 A23, SLC25 A43, SLC35 A2, SLC35 C1* and *SLC48 A1* were downregulated in HM embryos (Table S2). Interestingly, including 33 downregulated genes in HM, (or conversely upregulated in HF embryos), 17.3% of the DEGs were located on the X chromosome (Figure S3B). Gene enrichment analysis revealed that genes that showed sexually dimorphic expression exclusively in embryos developed in heifers were involved in various biological processes, including female sex differentiation, developmental processes (in placenta development, nervous system development, blood vessel development, epithelium development and differentiation), and cell adhesion (Fig. 6).

### Sexually dimorphic gene expression patterns in day 13 embryos irrespective of the maternal reproductive tract environment

Genes that showed sexually dimorphic expression in embryos irrespective of the maternal environment were identified by filtering those that exhibited sexually dimorphic expression in both cow and heifer embryos using Venny 2.1 software. Accordingly, only 38 genes were commonly differentially expressed in CM vs. CF as well as in HM vs. HF (Fig. 6). Of these, 7 genes (*IRS4*, *PLS3, SCN3 A, NUDT10, SLC30 A10, SLC10 A4* and *ARHGEF10)* were upregulated and 31 genes including *DHRS9, TKDP4, TKDP1, CDKN1 C, SRARP* and *TBX2,* were downregulated in CM compared to CF as well as in HM compared to HF embryos (Table 5). Furthermore, the chromosomal analysis revealed that 18 of the 38 DEGs were located on the X chromosome, and 16 DEGs located on the X chromosome were upregulated in female embryos.

**Table 5:** The upregulated (↑) or downregulated (↓) genes in both CM vs. CF and HM vs. HF comparisons

			CM vs. CF			HM vs. HF		
Gene symbol	Ensembl gene id	Chr	P value	FDR	Expression patterns	P value	FDR	Expression patterns
*IRS4*	ENSBTAG00000006535	X	< 0.001	0.025	↑	< 0.001	0.021	↑
*PLS3*	ENSBTAG00000011613	X	< 0.001	0.000	↑	< 0.001	0.000	↑
*SCN3 A*	ENSBTAG00000019385	2	0.001	0.094	↑	< 0.001	0.003	↑
*SLC30 A10*	ENSBTAG00000004414	16	< 0.001	0.014	↑	0.001	0.097	↑
*NUDT10*	ENSBTAG00000054383	X	< 0.001	0.060	↑	< 0.001	0.001	↑
*ARHGEF10*	ENSBTAG00000008585	27	< 0.001	0.068	↑	0.001	0.093	↑
*SLC10 A4*	ENSBTAG00000004888	6	< 0.001	0.015	↑	< 0.001	0.024	↑
*TIMM8 A*	ENSBTAG00000022292	X	< 0.001	0.018	↓	0.001	0.091	↓
*ATP6 AP1*	ENSBTAG00000012117	X	< 0.001	0.013	↓	< 0.001	0.042	↓
*KDM5 C*	ENSBTAG00000014943	X	< 0.001	0.003	↓	< 0.001	0.025	↓
*DNASE1L1*	ENSBTAG00000007455	X	< 0.001	0.061	↓	< 0.001	0.012	↓
*ENSBTAG00000022275*	ENSBTAG00000022275	1	< 0.001	0.001	↓	< 0.001	0.008	↓
*MOSPD2*	ENSBTAG00000018292	X	< 0.001	0.087	↓	< 0.001	0.024	↓
*RAB9 A*	ENSBTAG00000010923	X	< 0.001	0.001	↓	< 0.001	0.049	↓
*ENSBTAG00000005239*	ENSBTAG00000005239	2	< 0.001	0.001	↓	< 0.001	0.049	↓
*TRAPPC2*	ENSBTAG00000010922	X	< 0.001	0.010	↓	< 0.001	0.001	↓
*ATP2 C2*	ENSBTAG00000000945	18	0.001	0.081	↓	< 0.001	0.060	↓
*RAB33 A*	ENSBTAG00000034712	X	< 0.001	0.058	↓	< 0.001	0.002	↓
*IKBKG*	ENSBTAG00000006268	X	< 0.001	0.000	↓	< 0.001	0.014	↓
*RNF113 A*	ENSBTAG00000006034	X	< 0.001	0.014	↓	< 0.001	0.016	↓
*ABHD4*	ENSBTAG00000016658	10	< 0.001	0.007	↓	< 0.001	0.035	↓
*LAS1L*	ENSBTAG00000002210	X	< 0.001	0.000	↓	0.001	0.065	↓
*MPP1*	ENSBTAG00000013046	X	< 0.001	0.000	↓	0.001	0.073	↓
*PIGA*	ENSBTAG00000007646	X	< 0.001	0.082	↓	0.001	0.073	↓
*ENSBTAG00000052866*	ENSBTAG00000052866	3	< 0.001	0.044	↓	0.001	0.087	↓
*SYN1*	ENSBTAG00000005042	X	< 0.001	0.020	↓	< 0.001	0.029	↓
*PRXL2 A*	ENSBTAG00000021416	28	< 0.001	0.057	↓	0.001	0.075	↓
*HAND1*	ENSBTAG00000002335	7	< 0.001	0.016	↓	< 0.001	0.000	↓
*TKDP1*	ENSBTAG00000018122	13	< 0.001	0.022	↓	< 0.001	0.009	↓
*TBX2*	ENSBTAG00000014278	19	< 0.001	0.010	↓	< 0.001	0.000	↓
*TMEM102*	ENSBTAG00000031737	19	< 0.001	0.000	↓	< 0.001	0.000	↓
*ABCG2*	ENSBTAG00000017704	6	< 0.001	0.027	↓	< 0.001	0.037	↓
*SRARP*	ENSBTAG00000010938	2	< 0.001	0.003	↓	< 0.001	0.006	↓
*PINLYP*	ENSBTAG00000001260	18	< 0.001	0.018	↓	< 0.001	0.003	↓
*CDKN1 C*	ENSBTAG00000031184	29	< 0.001	0.013	↓	0.000	0.003	↓
*TKDP4*	ENSBTAG00000013950	13	< 0.001	0.003	↓	0.000	0.000	↓
*C15H11orf96*	ENSBTAG00000045822	15	< 0.001	0.001	↓	0.000	0.023	↓
*DHRS9*	ENSBTAG00000004557	2	< 0.001	0.002	↓	0.000	0.013	↓

## Discussion

### Sexually dimorphic gene expression in day 13 embryos was influenced by the maternal environment

In the current study, the sexually dimorphic gene expression in embryos developed in lactating cows and nonlactating nulliparous heifers on day 13 of gestation was investigated. Although the metabolic status of these animals was not explicitly examined, it is reasonable to assume that these groups of animals differed at least in their metabolic status. Nonlactating heifers represent nearly an ideal reproductive tract environment for embryo development, whereas lactating cows serve as a model of an unfavourable and metabolically demanding maternal environment. Thus, a detailed understanding of the molecular responses of male and female embryos to the reproductive tract microenvironment of lactating cows and nonlactating nulliparous heifers provides insights into sex-specific developmental programming that occurs due to differences in developmental conditions. Accordingly, in the current study, while 159 and 254 genes showed sexually dimorphic expression exclusively in embryos developed in lactating cows and nonlactating nulliparous heifers, respectively, only 38 genes showed sexually dimorphic expression patterns in both embryo groups (Fig. 6). These results suggest that the sexually dimorphic gene expression in day 13 embryos may be influenced more by the maternal environment (developmental conditions) than by intrinsic gender differences. However, further study may be needed to confirm this claim.

It may be possible to speculate that genes that showed sexually dimorphic expression exclusively in embryos developed in heifers represent a set of genes that normally show differential expression patterns between male and female embryos when development occurs in a favourable environment. Meanwhile, those genes that showed sexually dimorphic expression patterns exclusively in embryos developed in cows may represent a set of genes whose expression in male and female embryos is influenced by suboptimal developmental conditions. Consistent with these findings, a previous study also identified sexually dimorphic expression of 254, 54 and 37 genes in embryos developed in vivo, serum-containing media, and serum-free in vitro culture media, respectively [15]. This may also indicate that sexually dimorphic gene expression in embryos may be regulated more by developmental (culture) conditions than by the sex of the embryo. Moreover, a comparative analysis of our results, specifically those obtained from embryos developed in heifers, with the results reported by Forde et al. [18], who investigated the sexually dimorphic expression in day 19 conceptuses developed in crossbred beef heifers, indicated that both studies had 21 genes in common. Among these, 4 genes including *ADAMTS19* were upregulated and 8 genes including *DLX3, SLC10 A3,* and *SLC35 C1* were downregulated in male embryos (Table 6), suggesting that the sexually dimorphic expression trend of these genes may remain similar both in day 13 and day 19 bovine embryos.

**Table 6:** Upregulated or downregulated genes in male compared to female embryos in both day 13 and day 19 embryos

Ensembl gene id	Gene symbol	Gene description	Chr	Male vs. Female embryos	
				Current study	Forde et al*.* [18]
ENSBTAG00000011613	*PLS3*	Plastin 3	X	↑	↑
ENSBTAG00000054383	*NUDT10*	Nudix (nucleoside diphosphate linked moiety X)-type motif 10	X	↑	↑
ENSBTAG00000033803	*FABP7*	fatty acid binding protein 7	9	↑	↑
ENSBTAG00000016145	*ADAMTS19*	ADAM metallopeptidase with thrombospondin type 1 motif 19	7	↑	↑
ENSBTAG00000010587	*SH3BGRL*	SH3 domain binding glutamate rich protein like	X	↑	↑
ENSBTAG00000023652	*PROS1*	protein S	1	↑	↑
ENSBTAG00000017409	*DLX3*	Distal-less homeobox 3	19	↓	↓
ENSBTAG00000016085	*IRAK1*	Interleukin 1 receptor associated kinase 1	X	↓	↓
ENSBTAG00000012117	*ATP6 AP1*	ATPase H + transporting accessory protein 1	X	↓	↓
ENSBTAG00000019061	*ELF4*	E74 like ETS transcription factor 4	X	↓	↓
ENSBTAG00000014333	*SLC10 A3*	Solute carrier family 10 member 3	X	↓	↓
ENSBTAG00000005042	*SYN1*	Synapsin I	X	↓	↓
ENSBTAG00000048365	*WNT7B*	Wnt family member 7B	5	↓	↓
ENSBTAG00000003199	*SLC35 C1*	Solute carrier family 35 member C1	15	↓	↓
ENSBTAG00000031737	*TMEM102*	Transmembrane protein 102	19	↓	↓
ENSBTAG00000017690	*CARNS1*	Carnosine synthase 1	29	↓	↓
ENSBTAG00000015273	*CAND2*	Cullin associated and neddylation dissociated 2 (putative)	22	↓	↓
ENSBTAG00000045989	*CDC42EP5*	CDC42 effector protein 5	18	↑	↓
ENSBTAG00000002683	*PFKP*	Phosphofructokinase, platelet	13	↑	↓
ENSBTAG00000015630	*RLIM*	Ring finger protein, LIM domain interacting	X	↓	↑
ENSBTAG00000017258	*ACSL3*	Acyl-CoA synthetase long chain family member 3	2	↓	↑
ENSBTAG00000022292	*TIMM8 A*	Translocase of inner mitochondrial membrane 8 A	X	↓	↑
ENSBTAG00000006307	*HSD17B11*	Hydroxysteroid 17-beta dehydrogenase 11	6	↓	↑
ENSBTAG00000013957	*GALNT3*	Polypeptide N- acetylgalactosaminyltransferase 3	2	↓	↑
ENSBTAG00000021751	*RASEF*	RAS and EF-hand domain containing	8	↓	↑
ENSBTAG00000007646	*PIGA*	Phosphatidylinositol glycan anchor biosynthesis class A	X	↓	↑
ENSBTAG00000050091	*GCNT4*	Glucosaminyl (N-acetyl) transferase 4	10	↓	↑
ENSBTAG00000016805	*SGMS2*	Sphingomyelin synthase 2	6	↓	↑

*Symbols ↑ and ↓ indicate increased and decreased gene expression levels in male compared to female embryos, respectively*

Interestingly, in the current study, 31.2% and 17.3% of the genes that showed sexually dimorphic expression exclusively in embryos developed in cows and heifers respectively, were located on the X chromosome (Figure S3 A & Figure S3B), and when all genes that showed sexually dimorphic expression in embryos of cow and heifers were considered, these proportions increased to 36% and 21%, respectively. Previous studies also reported that 2.7% (*n* = 139) of genes that showed sexually dimorphic expression in day 19 of bovine embryos [18] and 7.1% (*n* = 163) of genes that showed sexually dimorphic expression in the blastocysts developed in SOF media supplemented with 5% FCS [11] were located on the X chromosome. In our study, 96% and 75% of the genes on the X chromosome which showed sexually dimorphic expression in cow and heifer embryos, respectively, were upregulated in female embryos (Table S1, Table S2). This phenomenon may be partly related to incomplete X chromosome inactivation in female embryos. The presence of a double X chromosome in females could lead to sexual dimorphism by affecting the transcriptional levels of genes encoded by sex chromosomes and/or autosomal chromosomes [11]. Thus, based on the results of the current study, it could be speculated that X chromosome inactivation was not yet fully completed at the initiation of elongation around day 13 in bovine embryos. Moreover, the results of the present study may suggest that X chromosome inactivation might differ depending on the developmental environment. Considering the higher proportion of genes that exhibited sexually dimorphic expression on the X chromosome in embryos developed in lactating cows compared to those developed in heifers, it can be suggested that X chromosome inactivation in embryos developed in cows may be less effective compared to those developed in heifers. The effect of environmental conditions on the dosage compensation of X-linked genes as evidenced in bovine female preimplantation embryos [16] and the impaired X-chromosome inactivation due to suboptimal developmental conditions may affect the embryo viability leading to skewed sex ratios [33].

### Genes that showed sexually dimorphic expression patterns in day 13 embryos due to reproductive tract environment lactating cows were involved in steroid biosynthesis and metabolism

In addition to examining the genes that showed sexually dimorphic expression patterns in day 13 embryos, we also investigated the function of those genes by performing gene ontology enrichment analysis and literature mining. Accordingly, we identified key functional differences in embryos developed in cows or developed in heifers. For instance, steroid biosynthesis and metabolism was one of the biological processes enriched by genes that showed sexually dimorphic expression exclusively in embryos developed in lactating cows (Figs. 3 and 6). These genes include the aromatases (*CYP19 A1), HSD17B1, HSD17B10, CYP39 A1, CYP2R1* and *CYP27B1* (Fig. 3*)*. Previous studies indicated that aromatase (*CYP19 A1*), which is involved in the conversion of androgens to estradiol [34], showed female-specific expression patterns during bovine pregnancy [35] and this gene is required for sex differentiation during embryonic development [36]. Similarly, *HSD17B1* is involved in the conversion of estrone and estradiol or androstenedione and testosterone or vice-versa [37–39]. In fact, steroids are involved in embryo growth, embryo-maternal signalling and communication [40] and successful pregnancy establishment, see review [41]. The amount and availability of steroid hormones during pregnancy can be modulated by factors such as stress, which can ultimately lead to a reduction in various hormones during pregnancy [42]. This may be more relevant in lactating postpartum cows, as many high-yielding dairy cows may experience metabolic stress during early lactation. During the early stages of lactation, the majority of high yielding dairy cows enter to negative energy balance (NEB) and during this period, various metabolites including ketone bodies, ß-hydroxybutyrate and non-esterified fatty acids (NEFA) can be released into the circulation of the cow [43]. Higher concentrations of these metabolites may ultimately disturb the microenvironment of the reproductive tract and greatly affect the expression of steroidogenesis genes in embryos. Therefore, sexually dimorphic expression of key genes which were involved in steroidogenesis only in embryos developed in cows may provide a clue about the presence of sexual dimorphism in the steroidogenesis activity of cow embryos.

### Genes that showed sexually dimorphic expression patterns in day 13 embryos due to the lactating reproductive tract environment of cows were involved in the gastrulation

Gastrulation is believed to be a key step in the process of embryo development. In bovine, the embryonic ectoderm appears around day 13 of gestation followed by the formation of two additional layers (mesoderm and endoderm) by days 14–15 [44]. In line with this, in the current study genes that were involved in gastrulation, namely *TBX6*, *DUSP1, GJA, HMGA2,* and *COL12 A1* showed sexually dimorphic expression patterns only in embryos developed in cows but not in embryos developed in heifers (Fig. 3, Table S1). The first two genes were upregulated in the male embryos and the latter three were upregulated in the female embryos. Apart from gene enrichment analysis, results from previous studies have also highlighted the role of these genes in gastrulation processes. For instance, the importance of T-box transcription factor 6 (*TBX6*) in mesoderm specification and function [45–47], *DUSP1* in germ layer specification [48], and gap junction protein alpha 1 (*GJA1*), also known as connexin 43 (*CX43*), in gastrulation processes [49] have been described. Thus, sexually dimorphic expression of these genes exclusively in embryos developed in cows may suggest the presence of sex specific gastrulation activities by male and female embryos in a cow’s reproductive tract environments. Indeed, earlier studies in mice have reported that the duration of in vitro culture strongly affected X chromosome inactivation in the primitive endoderm of mice [50]. Although caution should be taken, this may also indicate the existence of a direct link between developmental environment conditions and the successful germ layer establishment during the gastrulation processes.

### Genes that showed sexually dimorphic expression patterns in day 13 embryos due to the reproductive tract environment of heifers were associated with female sex differentiation

Sexual differentiation is one of the fundamental developmental processes that lead to the development of male or female phenotypes from undifferentiated embryonic structures [51]. This process is governed by sex-specific actions of gene networks, ultimately resulting in the conversion of the bipotential gonads of the growing fetus into either testis or ovaries [52]. In the current study, some genes associated with female sex differentiation, namely, *FZD4, FOXO3, BMPR1B, CEBPB, PTGS2* and *LHFPL2* showed sexually dimorphic expression patterns only in embryos developed in heifers (Fig. 5, Table S2). These genes except *BMPR1B* were downregulated in male embryos. Previous studies also indicated the role of frizzled class receptor 4 (*FZD4)* in gonad differentiation [53], forkhead box O3 *(FOXO3)* in ovarian development [54], bone morphogenetic protein receptor type 1B *(BMPR1B)* in germ-cell differentiation [55]*,* and LHFPL tetraspan subfamily member 2 (*LHFPL2*) in distal reproductive tract development [56]*.* Thus, the sexually dimorphic expression of genes associated with female sex differentiation in the embryos of heifers may indicate normal gonadal differentiation programming in developing embryos. In fact, these genes did not show sexually dimorphic expression patterns in embryos developed in cows, which may be affected by lactation and metabolic stress. However, we cannot rule out the presence of normal sexual gonadal differentiation in embryos developed in cows, as we have no experimental evidence to support the critical role of these genes on sex differentiation in bovine. Since the current study was limited to day 13 of gestation, further studies may be necessary to investigate the crucial role of these genes in gonadal differentiation and embryo development beyond day 13 of gestation.

### Genes that showed sexually dimorphic expression patterns in day 13 embryos due to the reproductive tract environment of heifers were involved in transmembrane transport

Several genes associated with transmembrane transport, such as *GLUT4* (*SLC2 A4*) and ten solute carrier gene families (*SLC16 A9, SLC10 A1, SLC10 A3, SLC16 A5, SLC22 A23, SLC25 A43, SLC35 A2, SLC35 C1, SLC38 A11* and *SLC48 A1)* showed sexually dimorphic expression patterns exclusively in embryos developed in heifers (Fig. 5, Table S2). Indeed, the amount and type of solutes that enter and leave a cell are regulated by the complex interactions between the membrane and macromolecules such as carbohydrates, proteins, and lipids [57]. For instance, *SLC2 A4* (*GLUT4*) is involved in glucose transport [58], glucose uptake, and glucose homeostasis [59]. Malfunctioning of *GLUT4* is believed to impair embryo implantation [60].

Apart from *GLUT4*, solute carriers involved in monocarboxylate cotransporter (*SLC16 A9*) [61], sodium bile salt cotransport (*SLC10 A1* & *SLC10 A3*) [62], monocarboxylate transport (*SLC16 A5*) [61], molecule transport across the mitochondria membrane (*SLC22 A23* & *SLC25 A43*) [63], nucleotide sugars transport *(SLC35 A2, SLC35 C1*) [64], sodium-coupled neutral amino acids transport (*SLC38 A11*) [65], and heme transport (*SLC48 A1*) [65, 66]], showed sexually dimorphic expression patterns only in embryos developed in heifers. Interestingly, with the exception of two genes (*SLC2 A4* and *SLC38 A11)*, all those solute carriers were downregulated in male embryos suggesting that male and female embryos seem to respond to the reproductive tract environment by expressing genes associated with memebrane transport in a sex specific manner.

Similarly, gene set enrichment analysis indicated that genes that exhibited sexually dimorphic expression patterns exclusively in embryos developed in heifers, namely CCAAT/enhancer-binding protein beta (*CEBPB),* prostaglandin-endoperoxide synthase *(PTGS2)*, distal-less homeobox 3 (*DLX3)*, and NAD(P) dependent steroid dehydrogenase-like (*NSDHL),* were involved in placenta development. Literature mining also indicated that *CEBPB*, one of the key genes expressed in trophoblasts, is involved in extravillous trophoblasts function [67] and *DLX3,* a homeodomain-containing transcription factor, is involved in normal placental morphogenesis [68] and regulation of villous cytotrophoblast differentiation [69]. Similarly, the X-linked genes, *NSDHL* and COX- 2 (*PTGS2*), are also associated with normal placental development [70, 71].

### Genes that showed sex specific expression patterns in day 13 embryos irrespective of the maternal environment

In addition to genes that showed sexually dimorphic expression patterns exclusively in embryos developed in cows and heifers, we have also sought to identify genes that showed sexually dimorphic expression patterns due to the sex of the embryos. In theory, it appears challenging to identify genes that are inherently regulated by the sex of the embryo or by the developmental conditions. Since the present study contrasted two maternal environments and two sexes, the experimental design feasibly identifies genes that exhibit sexually dimorphic expression patterns either due to the maternal environment or embryo sex. In fact, in the absence of the influence of the maternal environment, one could expect the same genes to show sexually dimorphic expression patterns in both embryos developed in cows and embryos developed in heifers. However, the current study found only 38 genes that showed sexually dimorphic expression patterns in embryos of cows and heifers (Fig. 6, Table 5). These genes represented only 19% and 13% of all genes that exhibited sexually dimorphic expression patterns in embryos of cows and heifers, respectively. These genes may represent a set of highly conserved sexually dimorphic genes whose expression in embryos is not affected by the maternal environment. We speculate that the expression patterns of these genes in embryos depend on the sex of the embryo rather than on the maternal environment. Besides, the expression trends of these genes showed that 31 out of genes including *IRS4*, *PIGA*, *TRAPPC2* and *KDM5 C* were upregulated in the female embryos of cows and heifers and 16 out of 38 genes (42.1%) were located on the X-chromosome.

## Conclusion

In the current, while 159 and 254 genes showed sexually dimorphic expression patterns exclusively in embryos developed in cows and heifers, respectively, only 38 genes showed sexually dimorphic expression patterns in both cow and heifer embryos. Genes that showed sexually dimorphic expression patterns in both cow and heifer embryos may represent genes regulated by the sex of the embryos not by the maternal reproductive tract microenvironment. Genes that showed sexually dimorphic expression patterns exclusively in embryos developed in lactating cows may represent those genes whose expression may be altered in male or female embryos due to a suboptimal maternal environment. These genes were involved in various functions including steroid biosynthesis and gastrulation. On the other hand, genes that showed sexually dimorphic expression patterns only in embryos developed in heifers may represent gene sets that can be expressed differently in male and female embryos only when development takes place in a favourable environment. These genes were involved in various functions including female sex differentiation, placental development and transmembrane transport. In addition, a higher proportion of genes that showed sexually dimorphic expression patterns in cow embryos were located on the X chromosome and the majority of these were upregulated in female embryos. Overall, the current study identified several genes that exhibit sexually dimorphic expression patterns in day 13 bovine embryos as a result of the maternal reproductive tract microenvironment or solely due to the embryo sex.

## Supplementary Information

Supplementary Material 1.

## Data Availability

The datasets generated and/or analysed during the current study are available in the NCBI repository, https://www.ncbi.nlm.nih.gov/geo/query/acc.cgi?acc=GSE276275.

## Abbreviations

CM: Day 13 male embryos developed in cows
CF: Day 13 female embryos developed in cows
HM: Day 13 male embryos developed in heifers
HF: Day 13 female embryos developed in heifers

## References

[1] Avery B. Impact of asynchronous ovulations on the expression of sex-dependent growth rate in bovine preimplantation embryos. J Reprod Fertil. 1989;87:627–31. 10.1530/jrf.0.0870627. 2600913 10.1530/jrf.0.0870627

[2] Avery B, Jørgensen CB, Madison V, Greve T. Morphological development and sex of bovine in vitro-fertilized embryos. Mol Reprod Dev. 1992;32:265–70. 10.1002/mrd.1080320312. 1497876 10.1002/mrd.1080320312

[3] Avery B, Madison V, Greve T. Sex and development in bovine in-vitro fertilized embryos. Theriogenology. 1991;35:953–63. 10.1016/0093-691x(91)90306-x. 16726963 10.1016/0093-691x(91)90306-x

[4] Gutiérrez-Adán A, Oter M, Martínez-Madrid B, Pintado B, La Fuente J, de. Differential expression of two genes located on the X chromosome between male and female in vitro-produced bovine embryos at the blastocyst stage. Mol Reprod Dev. 2000;55:146–51. 10.1002/(SICI)1098-2795(200002)55:2%3c146::AID-MRD3%3e3.0.CO;2-F. 10618653 10.1002/(SICI)1098-2795(200002)55:2<146::AID-MRD3>3.0.CO;2-F

[5] Tiffin GJ, Rieger D, Betteridge KJ, Yadav BR, King WA. Glucose and glutamine metabolism in pre-attachment cattle embryos in relation to sex and stage of development. J Reprod Fertil. 1991;93:125–32. 10.1530/jrf.0.0930125. 1920281 10.1530/jrf.0.0930125

[6] Bredbacka K, Bredbacka P. Glucose controls sex-related growth rate differences of bovine embryos produced in vitro. J Reprod Fertil. 1996;106:169–72. 10.1530/jrf.0.1060169. 8699398 10.1530/jrf.0.1060169

[7] Larson MA, Kimura K, Kubisch HM, Roberts RM. Sexual dimorphism among bovine embryos in their ability to make the transition to expanded blastocyst and in the expression of the signaling molecule IFN-tau. Proc Natl Acad Sci U S A. 2001;98:9677–82. 10.1073/pnas.171305398. 11481449 10.1073/pnas.171305398 PMC55511

[8] Gutiérrez-Adán A, Granados J, Pintado B, La Fuente J, de. Influence of glucose on the sex ratio of bovine IVM/IVF embryos cultured in vitro. Reprod Fertil Dev. 2001;13:361–5. 10.1071/rd00039. 11833931 10.1071/rd00039

[9] Dallemagne M, Ghys E, de Schrevel C, Mwema A, de Troy D, Rasse C, Donnay I. Oxidative stress differentially impacts male and female bovine embryos depending on the culture medium and the stress condition. Theriogenology. 2018;117:49–56. 10.1016/j.theriogenology.2018.05.020. 29859336 10.1016/j.theriogenology.2018.05.020

[10] Taqi MO, Saeed-Zidane M, Gebremedhn S, Salilew-Wondim D, Khdrawy O, Rings F, Neuhoff C, Hoelker M, Schellander K, Tesfaye D. Sexual dimorphic expression and release of transcription factors in bovine embryos exposed to oxidative stress. Mol Reprod Dev. 2019;86:2005–19. 10.1002/mrd.23272. 31544319 10.1002/mrd.23272

[11] Bermejo-Alvarez P, Rizos D, Rath D, Lonergan P, Gutierrez-Adan A. Sex determines the expression level of one third of the actively expressed genes in bovine blastocysts. Proc Natl Acad Sci U S A. 2010;107:3394–9. 10.1073/pnas.0913843107. 20133684 10.1073/pnas.0913843107 PMC2840439

[12] Kobayashi S, Isotani A, Mise N, Yamamoto M, Fujihara Y, Kaseda K, Nakanishi T, Ikawa M, Hamada H, Abe K, Okabe M. Comparison of gene expression in male and female mouse blastocysts revealed imprinting of the X-linked gene, Rhox5/Pem, at preimplantation stages. Curr Biol. 2006;16:166–72. 10.1016/j.cub.2005.11.071. 16431368 10.1016/j.cub.2005.11.071

[13] Kobayashi S, Fujihara Y, Mise N, Kaseda K, Abe K, Ishino F, Okabe M. The X-linked imprinted gene family Fthl17 shows predominantly female expression following the two-cell stage in mouse embryos. Nucleic Acids Res. 2010;38:3672–81. 10.1093/nar/gkq113. 20185572 10.1093/nar/gkq113 PMC2887969

[14] Denicol AC, Leão BCS, Dobbs KB, Mingoti GZ, Hansen PJ. Influence of Sex on Basal and Dickkopf-1 Regulated Gene Expression in the Bovine Morula. PLoS ONE. 2015;10: e0133587. 10.1371/journal.pone.0133587. 26196299 10.1371/journal.pone.0133587 PMC4510475

[15] Heras S, De Coninck, Dieter I M, van Poucke M, Goossens K, Bogado Pascottini O, van Nieuwerburgh F, Deforce D, Sutter P de, Leroy, Jo L M R, Gutierrez-Adan A, Peelman L, van Soom A. Suboptimal culture conditions induce more deviations in gene expression in male than female bovine blastocysts. BMC Genomics. 2016;17:72. 10.1186/s12864-016-2393-z. 10.1186/s12864-016-2393-z PMC4724126 26801242

[16] Wrenzycki C, Lucas-Hahn A, Herrmann D, Lemme E, Korsawe K, Niemann H. In vitro production and nuclear transfer affect dosage compensation of the X-linked gene transcripts G6PD, PGK, and Xist in preimplantation bovine embryos. Biol Reprod. 2002;66:127–34. 10.1095/biolreprod66.1.127. 11751274 10.1095/biolreprod66.1.127

[17] Morton KM, Herrmann D, Sieg B, Struckmann C, Maxwell WMC, Rath D, Evans G, Lucas-Hahn A, Niemann H, Wrenzycki C. Altered mRNA expression patterns in bovine blastocysts after fertilisation in vitro using flow-cytometrically sex-sorted sperm. Mol Reprod Dev. 2007;74:931–40. 10.1002/mrd.20573. 17219418 10.1002/mrd.20573

[18] Forde N, Maillo V, O’Gaora P, Simintiras CA, Sturmey RG, Ealy AD, Spencer TE, Gutierrez-Adan A, Rizos D, Lonergan P. Sexually Dimorphic Gene Expression in Bovine Conceptuses at the Initiation of Implantation. Biol Reprod. 2016;95:92. 10.1095/biolreprod.116.139857. 27488033 10.1095/biolreprod.116.139857 PMC5333939

[19] Sánchez JM, Simintiras CA, Lonergan P. Aspects of embryo-maternal communication in establishment of pregnancy in cattle. Anim Reprod. 2019;16:376–85. 10.21451/1984-3143-AR2019-0075. 32435281 10.21451/1984-3143-AR2019-0075 PMC7234086

[20] Brooks K, Burns G, Spencer TE. Conceptus elongation in ruminants: roles of progesterone, prostaglandin, interferon tau and cortisol. J Anim Sci Biotechnol. 2014;5:53. 10.1186/2049-1891-5-53. 25810904 10.1186/2049-1891-5-53 PMC4373033

[21] Mathew DJ, Sánchez JM, Passaro C, Charpigny G, Behura SK, Spencer TE, Lonergan P. Interferon tau-dependent and independent effects of the bovine conceptus on the endometrial transcriptome†. Biol Reprod. 2019;100:365–80. 10.1093/biolre/ioy199. 30203055 10.1093/biolre/ioy199 PMC6378860

[22] Mathew DJ, Peterson KD, Senn LK, Oliver MA, Ealy AD. Ruminant conceptus-maternal interactions: interferon-tau and beyond. J Anim Sci. 2022. 10.1093/jas/skac123. 35772752 10.1093/jas/skac123 PMC9246669

[23] Clemente M, Lopez-Vidriero I, O’Gaora P, Mehta JP, Forde N, Gutierrez-Adan A, Lonergan P, Rizos D. Transcriptome changes at the initiation of elongation in the bovine conceptus. Biol Reprod. 2011;85:285–95. 10.1095/biolreprod.111.091587. 21508349 10.1095/biolreprod.111.091587

[24] Rizos D, Carter F, Besenfelder U, Havlicek V, Lonergan P. Contribution of the female reproductive tract to low fertility in postpartum lactating dairy cows. J Dairy Sci. 2010;93:1022–9. 10.3168/jds.2009-2605. 20172222 10.3168/jds.2009-2605

[25] Berg DK, van Leeuwen J, Beaumont S, Berg M, Pfeffer PL. Embryo loss in cattle between Days 7 and 16 of pregnancy. Theriogenology. 2010;73:250–60. 10.1016/j.theriogenology.2009.09.005. 19880168 10.1016/j.theriogenology.2009.09.005

[26] Betteridge KJ, Eaglesome MD, Randall GCB, Mitchell D. Collection, description and transfer of embryos from cattle 10–16 days after oestrus. Reproduction. 1980;59:205–16. 10.1530/jrf.0.0590205. 10.1530/jrf.0.0590205 7401037

[27] Pérez-Cerezales S, Ramos-Ibeas P, Rizos D, Lonergan P, Bermejo-Alvarez P, Gutiérrez-Adán A. Early sex-dependent differences in response to environmental stress. Reproduction. 2018;155:R39–51. 10.1530/REP-17-0466. 29030490 10.1530/REP-17-0466

[28] Salilew-Wondim D, Hoelker M, Held-Hoelker E, Rings F, Tholen E, Große-Brinkhaus C, Shellander K, Blaschka C, Besenfelder U, Havlicek V, Tesfaye D. Sexual dimorphic miRNA-mediated response of bovine elongated embryos to the maternal microenvironment. PLoS ONE. 2024;19: e0298835. 10.1371/journal.pone.0298835. 38422042 10.1371/journal.pone.0298835 PMC10903816

[29] Danecek P, Bonfield JK, Liddle J, Marshall J, Ohan V, Pollard MO, Whitwham A, Keane T, McCarthy SA, Davies RM, Li H. Twelve years of SAMtools and BCFtools. GigaScience. 2021;10:giab008. 10.1093/gigascience/giab008. 10.1093/gigascience/giab008 PMC7931819 33590861

[30] Robinson MD, McCarthy DJ, Smyth GK. edgeR: a Bioconductor package for differential expression analysis of digital gene expression data. Bioinformatics. 2010;26:139–40. 10.1093/bioinformatics/btp616. 19910308 10.1093/bioinformatics/btp616 PMC2796818

[31] Raudvere U, Kolberg L, Kuzmin I, Arak T, Adler P, Peterson H, Vilo J. g:Profiler: a web server for functional enrichment analysis and conversions of gene lists (2019 update). Nucleic Acids Res. 2019;47:W191–8. 10.1093/nar/gkz369. 31066453 10.1093/nar/gkz369 PMC6602461

[32] Shannon P, Markiel A, Ozier O, Baliga NS, Wang JT, Ramage D, Amin N, Schwikowski B, Ideker T. Cytoscape: a software environment for integrated models of biomolecular interaction networks. Genome Res. 2003;13:2498–504. 10.1101/gr.1239303. 14597658 10.1101/gr.1239303 PMC403769

[33] Tan K, An L, Miao K, Ren L, Hou Z, Tao L, Zhang Z, Wang X, Xia W, Liu J, Wang Z, Xi G, Gao S, Sui L, Zhu D-S, Wang S, Wu Z, Bach I, Chen D-B, Tian J. Impaired imprinted X chromosome inactivation is responsible for the skewed sex ratio following in vitro fertilization. Proc Natl Acad Sci U S A. 2016;113:3197–202. 10.1073/pnas.1523538113. 26951653 10.1073/pnas.1523538113 PMC4812732

[34] Chan HJ, Petrossian K, Chen S. Structural and functional characterization of aromatase, estrogen receptor, and their genes in endocrine-responsive and -resistant breast cancer cells. J Steroid Biochem Mol Biol. 2016;161:73–83. 10.1016/j.jsbmb.2015.07.018. 26277097 10.1016/j.jsbmb.2015.07.018 PMC4752924

[35] Ross DGF, Bowles J, Hope M, Lehnert S, Koopman P. Profiles of gonadal gene expression in the developing bovine embryo. Sex Dev. 2009;3:273–83. 10.1159/000252791. 19844082 10.1159/000252791

[36] Jin K, Zuo Q, Song J, Zhang Y, Chen G, Li B. CYP19A1 (aromatase) dominates female gonadal differentiation in chicken (Gallus gallus) embryos sexual differentiation. 2020. Biosci Rep. 10.1042/BSR20201576. 10.1042/BSR20201576 PMC7560524 32990306

[37] Järvensivu P, Saloniemi-Heinonen T, Awosanya M, Koskimies P, Saarinen N, Poutanen M. HSD17B1 expression enhances estrogen signaling stimulated by the low active estrone, evidenced by an estrogen responsive element-driven reporter gene in vivo. Chem Biol Interact. 2015;234:126–34. 10.1016/j.cbi.2015.01.008. 25617485 10.1016/j.cbi.2015.01.008

[38] Heinosalo T, Saarinen N, Poutanen M. Role of hydroxysteroid (17beta) dehydrogenase type 1 in reproductive tissues and hormone-dependent diseases. Mol Cell Endocrinol. 2019;489:9–31. 10.1016/j.mce.2018.08.004. 30149044 10.1016/j.mce.2018.08.004

[39] Saloniemi T, Jokela H, Strauss L, Pakarinen P, Poutanen M. The diversity of sex steroid action: novel functions of hydroxysteroid (17β) dehydrogenases as revealed by genetically modified mouse models. J Endocrinol. 2012;212:27–40. 10.1530/JOE-11-0315. 22045753 10.1530/JOE-11-0315

[40] Carson DD, Hsu Y-C, Lennarz WJ. Synthesis of steroids in postimplantation mouse embryos cultured in vitro. Dev Biol. 1982;91:402–12. 10.1016/0012-1606(82)90046-X. 7095272 10.1016/0012-1606(82)90046-x

[41] Morel Y, Roucher F, Plotton I, Goursaud C, Tardy V, Mallet D. Evolution of steroids during pregnancy: Maternal, placental and fetal synthesis. Ann Endocrinol. 2016;77:82–9. 10.1016/j.ando.2016.04.023. 10.1016/j.ando.2016.04.023 27155772

[42] Dimac-Stohl KA, Davies CS, Grebe NM, Stonehill AC, Greene LK, Mitchell J, Clutton-Brock T, Drea CM. Incidence and biomarkers of pregnancy, spontaneous abortion, and neonatal loss during an environmental stressor: Implications for female reproductive suppression in the cooperatively breeding meerkat. Physiol Behav. 2018;193:90–100. 10.1016/j.physbeh.2017.11.011. 29730033 10.1016/j.physbeh.2017.11.011

[43] McArt JAA, Nydam DV, Oetzel GR, Overton TR, Ospina PA. Elevated non-esterified fatty acids and β-hydroxybutyrate and their association with transition dairy cow performance. Vet J. 2013;198:560–70. 10.1016/j.tvjl.2013.08.011. 24054909 10.1016/j.tvjl.2013.08.011

[44] van Leeuwen J, Berg DK, Pfeffer PL. Morphological and Gene Expression Changes in Cattle Embryos from Hatched Blastocyst to Early Gastrulation Stages after Transfer of In Vitro Produced Embryos. PLoS ONE. 2015;10: e0129787. 10.1371/journal.pone.0129787. 26076128 10.1371/journal.pone.0129787 PMC4468082

[45] Chapman AB, Casida LE, Cote A. Sex ratios of fetal calves. J Anim Sci. 1938;1938:303–4. 10.2527/jas1938.19381303x.

[46] Chapman DL, Agulnik I, Hancock S, Silver LM, Papaioannou VE. Tbx6, a mouse T-Box gene implicated in paraxial mesoderm formation at gastrulation. Dev Biol. 1996;180:534–42. 10.1006/dbio.1996.0326. 8954725 10.1006/dbio.1996.0326

[47] Sadahiro T, Isomi M, Muraoka N, Kojima H, Haginiwa S, Kurotsu S, Tamura F, Tani H, Tohyama S, Fujita J, Miyoshi H, Kawamura Y, Goshima N, Iwasaki YW, Murano K, Saito K, Oda M, Andersen P, Kwon C, Uosaki H, Nishizono H, Fukuda K, Ieda M. Tbx6 Induces Nascent Mesoderm from Pluripotent Stem Cells and Temporally Controls Cardiac versus Somite Lineage Diversification. Cell Stem Cell. 2018;23:382-395.e5. 10.1016/j.stem.2018.07.001. 30100166 10.1016/j.stem.2018.07.001 PMC6190602

[48] Umair Z, Kumar S, Rafiq K, Kumar V, Reman ZU, Lee S-H, Kim S, Lee J-Y, Lee U, Kim J. Dusp1 modulates activin/smad2 mediated germ layer specification via FGF signal inhibition in Xenopus embryos. Anim Cells Syst (Seoul). 2020;24:359–70. 10.1080/19768354.2020.1847732. 33456720 10.1080/19768354.2020.1847732 PMC7782979

[49] Liptau H, Viebahn C. Expression patterns of gap junctional proteins connexin 32 and 43 suggest new communication compartments in the gastrulating rabbit embryo. Differentiation. 1999;65:209–19. 10.1046/j.1432-0436.1999.6540209.x. 10653357 10.1046/j.1432-0436.1999.6540209.x

[50] Fukuda A, Motosugi N, Ando M, Kimura M, Umezawa A, Akutsu H. Imprinted X-chromosome inactivation impacts primitive endoderm differentiation in mouse blastocysts. FEBS Lett. 2020;594:913–23. 10.1002/1873-3468.13676. 31721177 10.1002/1873-3468.13676

[51] Aatsha PA, Arbor TC, Krishan K. Embryology, Sexual Development in StatPearls. Treasure Island (FL): StatPearls; 2023. https://www.ncbi.nlm.nih.gov/books/NBK557601/. 32491533

[52] Reyes AP, León NY, Frost ER, Harley VR. Genetic control of typical and atypical sex development. Nat Rev Urol. 2023;20:434–51. 10.1038/s41585-023-00754-x. 37020056 10.1038/s41585-023-00754-x

[53] Piprek RP, Damulewicz M, Tassan J-P, Kloc M, Kubiak JZ. Transcriptome profiling reveals male- and female-specific gene expression pattern and novel gene candidates for the control of sex determination and gonad development in Xenopus laevis. Dev Genes Evol. 2019;229:53–72. 10.1007/s00427-019-00630-y. 30972573 10.1007/s00427-019-00630-y PMC6500517

[54] Carré G-A, Couty I, Hennequet-Antier C, Govoroun MS. Gene expression profiling reveals new potential players of gonad differentiation in the chicken embryo. PLoS ONE. 2011;6: e23959. 10.1371/journal.pone.0023959. 21931629 10.1371/journal.pone.0023959 PMC3170287

[55] Neumann JC, Chandler GL, Damoulis VA, Fustino NJ, Lillard K, Looijenga L, Margraf L, Rakheja D, Amatruda JF. Mutation in the type IB bone morphogenetic protein receptor Alk6b impairs germ-cell differentiation and causes germ-cell tumors in zebrafish. Proc Natl Acad Sci U S A. 2011;108:13153–8. 10.1073/pnas.1102311108. 21775673 10.1073/pnas.1102311108 PMC3156187

[56] Zhao F, Zhou J, Li R, Dudley EA, Ye X. Novel function of LHFPL2 in female and male distal reproductive tract development. Sci Rep. 2016;6:23037. 10.1038/srep23037. 26964900 10.1038/srep23037 PMC4786858

[57] Stillwell W. Chapter 19 - Membrane Transport. In: Stillwell W, editor. An Introduction to Biological Membranes (Second Edition): Elsevier; 2016. p. 423–451. 10.1016/B978-0-444-63772-7.00019-1.

[58] Uldry M, Thorens B. The SLC2 family of facilitated hexose and polyol transporters. Pflugers Archiv- Eur J Physiol. 2004;447:480–9. 10.1007/s00424-003-1085-0. 12750891 10.1007/s00424-003-1085-0

[59] Navarrete Santos A, Tonack S, Kirstein M, Kietz S, Fischer B. Two insulin-responsive glucose transporter isoforms and the insulin receptor are developmentally expressed in rabbit preimplantation embryos. Reproduction. 2004;128:503–16. 10.1530/rep.1.00203. 15509696 10.1530/rep.1.00203

[60] Long Y, Wang Y-C, Yuan D-Z, Dai X-H, Liao L-C, Zhang X-Q, Zhang L-X, Ma Y-D, Lei Y, Cui Z-H, Zhang J-H, Nie L, Yue L-M. GLUT4 in Mouse Endometrial Epithelium: Roles in Embryonic Development and Implantation. Front Physiol. 2021;12: 674924. 10.3389/fphys.2021.674924. 34248664 10.3389/fphys.2021.674924 PMC8267529

[61] Halestrap AP, Meredith D. The SLC16 gene family—from monocarboxylate transporters (MCTs) to aromatic amino acid transporters and beyond. Pflugers Archiv- Eur J Physiol. 2004;447:619–28. 10.1007/s00424-003-1067-2. 12739169 10.1007/s00424-003-1067-2

[62] Hagenbuch B, Dawson P. The sodium bile salt cotransport family SLC10. Pflugers ArchivEur- J Physiol. 2004;447:566–70. 10.1007/s00424-003-1130-z. 12851823 10.1007/s00424-003-1130-z

[63] Palmieri F. The mitochondrial transporter family (SLC25): physiological and pathological implications. Pflügers Archiv-Eur J Physiol. 2004;447:689–709. 10.1007/s00424-003-1099-7. 14598172 10.1007/s00424-003-1099-7

[64] Ishida N, Kawakita M. Molecular physiology and pathology of the nucleotide sugar transporter family (SLC35). Pflugers Archiv-Eur J Physiol. 2004;447:768–75. 10.1007/s00424-003-1093-0. 12759756 10.1007/s00424-003-1093-0

[65] Mackenzie B, Erickson JD. Sodium-coupled neutral amino acid (System N/A) transporters of the SLC38 gene family. Pflugers Archiv- Eur J Physiol. 2004;447:784–95. 10.1007/s00424-003-1117-9. 12845534 10.1007/s00424-003-1117-9

[66] White C, Yuan X, Schmidt PJ, Bresciani E, Samuel TK, Campagna D, Hall C, Bishop K, Calicchio ML, Lapierre A, Ward DM, Liu P, Fleming MD, Hamza I. HRG1 is essential for heme transport from the phagolysosome of macrophages during erythrophagocytosis. Cell Metab. 2013;17:261–70. 10.1016/j.cmet.2013.01.005. 23395172 10.1016/j.cmet.2013.01.005 PMC3582031

[67] Zhou W, Wang H, Yang Y, Guo F, Yu B, Su Z. Trophoblast Cell Subtypes and Dysfunction in the Placenta of Individuals with Preeclampsia Revealed by Single-Cell RNA Sequencing. Mol Cells. 2022;45:317–28. 10.14348/molcells.2021.0211. 35289305 10.14348/molcells.2021.0211 PMC9095508

[68] Morasso MI, Grinberg A, Robinson G, Sargent TD, Mahon KA. Placental failure in mice lacking the homeobox gene Dlx3. Proc Natl Acad Sci U S A. 1999;96:162–7. 10.1073/pnas.96.1.162. 9874789 10.1073/pnas.96.1.162 PMC15110

[69] Chui A, Evseenko DA, Brennecke SP, Keelan JA, Kalionis B, Murthi P. Homeobox gene Distal-less 3 (DLX3) is a regulator of villous cytotrophoblast differentiation. Placenta. 2011;32:745–51. 10.1016/j.placenta.2011.07.007. 21802725 10.1016/j.placenta.2011.07.007

[70] Caldas H, Cunningham D, Wang X, Jiang F, Humphries L, Kelley RI, Herman GE. Placental defects are associated with male lethality in bare patches and striated embryos deficient in the NAD(P)H Steroid Dehydrogenase-like (NSDHL) Enzyme. Mol Genet Metab. 2005;84:48–60. 10.1016/j.ymgme.2004.08.007. 15639195 10.1016/j.ymgme.2004.08.007

[71] Xu Y, Knipp GT, Cook TJ. Expression of Cyclooxygenase Isoforms in Developing Rat Placenta, Human Term Placenta, and BeWo Human Trophoblast Model. Mol Pharm. 2005;2:481–90. 10.1021/mp0500519. 16323955 10.1021/mp0500519

