# Supplementary material for: Sexually dimorphic gene expression responses of bovine embryos to the maternal microenvironment on day 13 of gestation
Source: BMC Genomics. 2025 Apr 14;26:372. doi: 10.1186/s12864-025-11570-5 (PMC11998263; doi:10.1186/s12864-025-11570-5)
Supplement: Supplementary file 1 — Supplementary Material 1. [file 12864_2025_11570_MOESM1_ESM.zip › Figure S2.pdf]

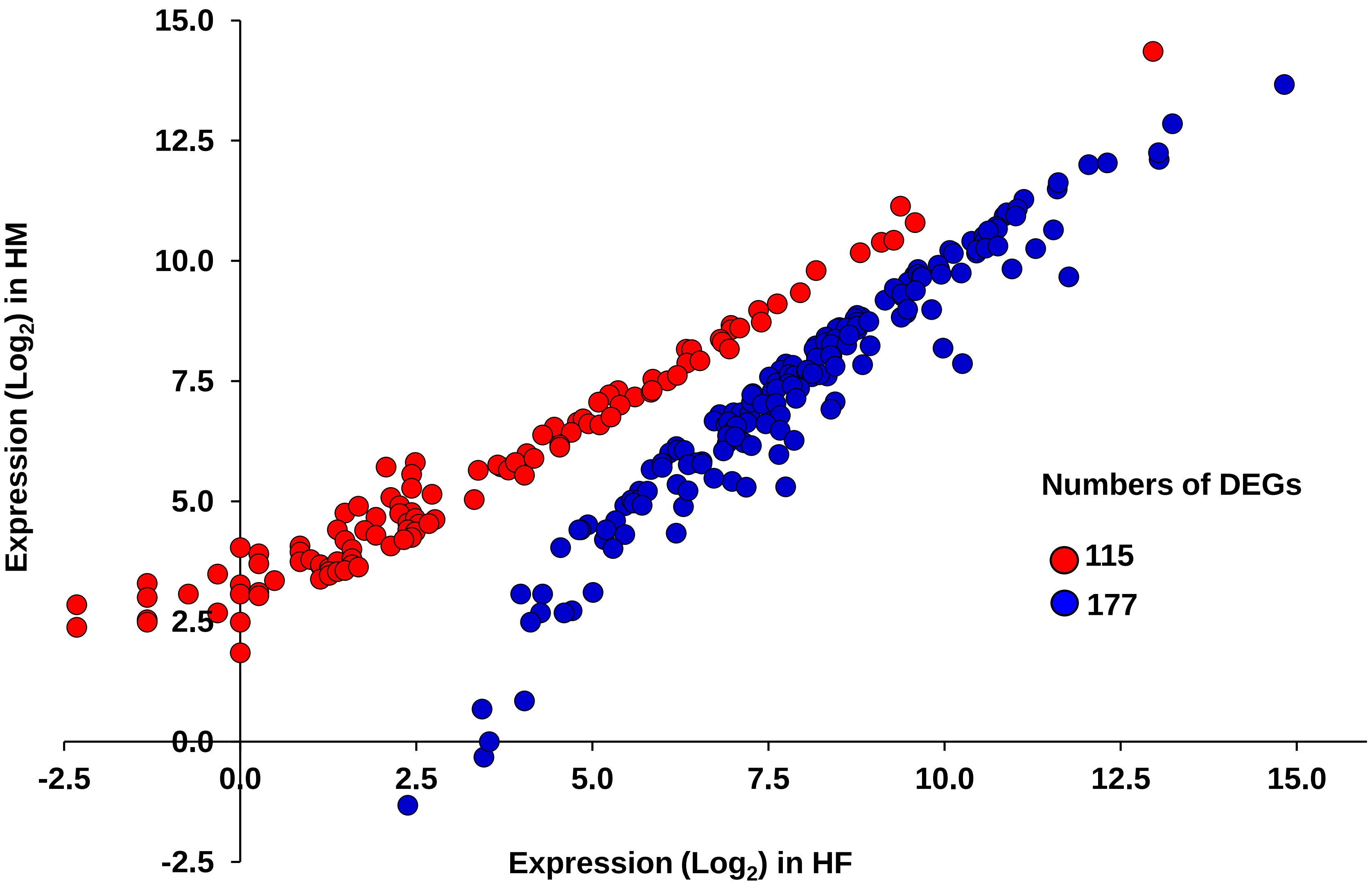

Figure S2 Expression patterns of the differentially expressed genes (DEGs) in (Log_2) in HM and HF embryos. Red and blue dots represent significantly upregulated and downregulated genes, respectively in HM compared to HF.
