# Supplementary material for: Sexually dimorphic gene expression responses of bovine embryos to the maternal microenvironment on day 13 of gestation
Source: BMC Genomics. 2025 Apr 14;26:372. doi: 10.1186/s12864-025-11570-5 (PMC11998263; doi:10.1186/s12864-025-11570-5)
Supplement: Supplementary file 1 — Supplementary Material 1. [file 12864_2025_11570_MOESM1_ESM.zip › Figure S3.pdf]

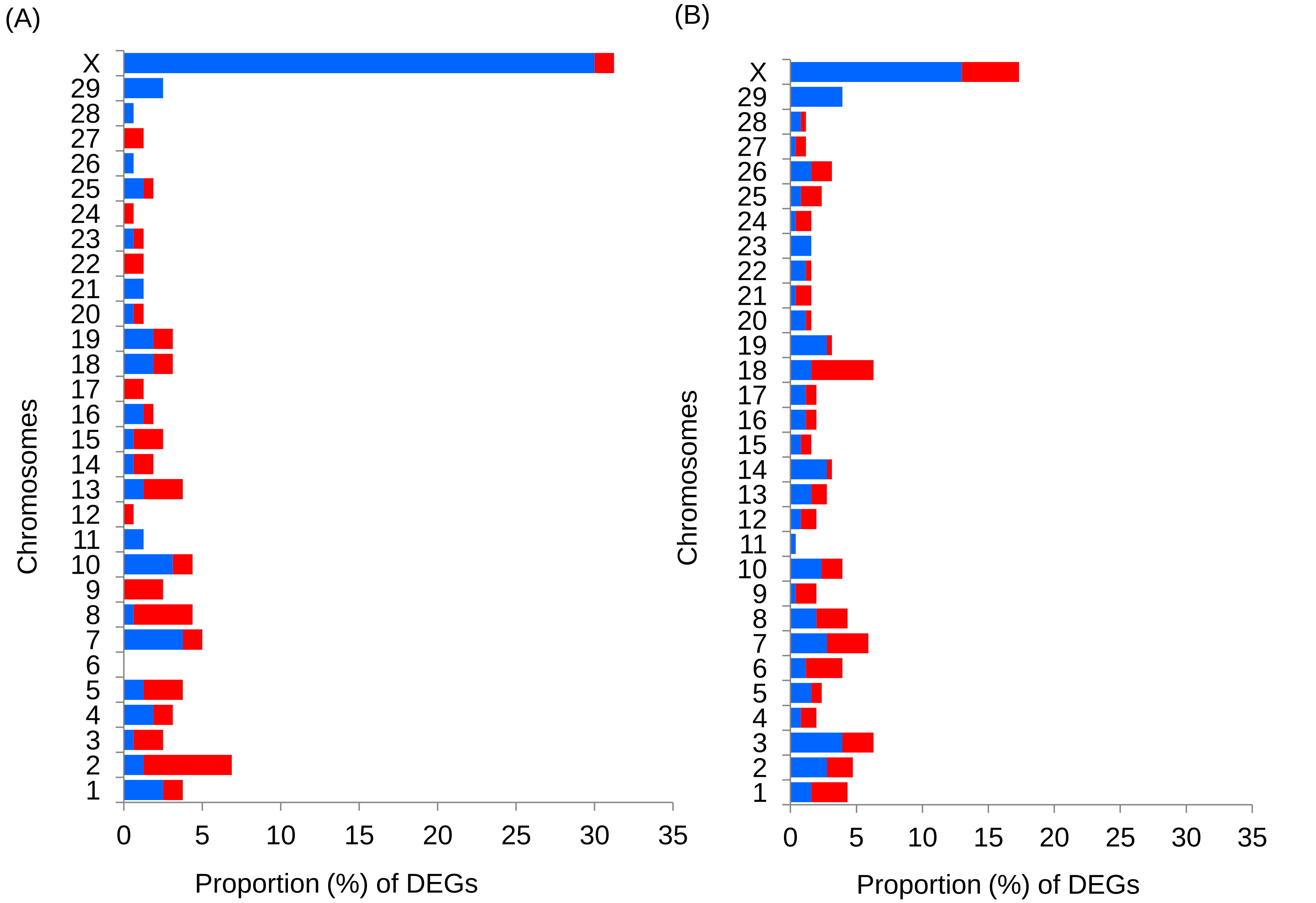

Figure S3 Chromosomal distribution of genes differentially expressed exclusively between CM and CF embryos (A) or between HM and HF embryos (B). Red and blue colors indicate the upregulated and downregulated DEGs, respectively, in CM compared to CF or in HM compared to HF.
